# The stochastic nature of errors in next-generation sequencing of circulating cell-free DNA

**DOI:** 10.1371/journal.pone.0229063

**Published:** 2020-02-21

**Authors:** David A. Nix, Sabine Hellwig, Christopher Conley, Alun Thomas, Carrie L. Fuertes, Cindy L. Hamil, Preetida J. Bhetariya, Ignacio Garrido-Laguna, Gabor T. Marth, Mary P. Bronner, Hunter R. Underhill

**Affiliations:** 1 Huntsman Cancer Institute, University of Utah School of Medicine, Salt Lake City, Utah, United States of America; 2 ARUP Laboratories, Salt Lake City, Utah, United States of America; 3 Divisions of Genetic Epidemiology and Public Health, Department of Family and Preventative Medicine, University of Utah, Salt Lake City, Utah, United States of America; 4 Department of Pathology, University of Utah, Salt Lake City, Utah, United States of America; 5 Department of Human Genetics, University of Utah, Salt Lake City, Utah, United States of America; 6 Division of Medical Genetics, Department of Pediatrics, University of Utah, Salt Lake City, Utah, United States of America; 7 Department of Radiology, University of Utah, Salt Lake City, Utah, United States of America; UCSI University, MALAYSIA

## Abstract

Challenges with distinguishing circulating tumor DNA (ctDNA) from next-generation sequencing (NGS) artifacts limits variant searches to established solid tumor mutations. Here we show early and random PCR errors are a principal source of NGS noise that persist despite duplex molecular barcoding, removal of artifacts due to clonal hematopoiesis of indeterminate potential, and suppression of patterned errors. We also demonstrate sample duplicates are necessary to eliminate the stochastic noise associated with NGS. Integration of sample duplicates into NGS analytics may broaden ctDNA applications by removing NGS-related errors that confound identification of true very low frequency variants during searches for ctDNA without *a priori* knowledge of specific mutations to target.

## Introduction

Cell-free DNA is an emerging molecular tool for non-invasive diagnosis and disease monitoring in a variety of human cancers [1]. Cell death is an on-going phenomenon that occurs in both healthy and cancerous tissues. As cells die, DNA released into the blood without a protective membrane is known as circulating cell-free DNA (ccfDNA). Mutations specific to a cancer are represented in the portion of ccfDNA derived from tumor cells and has been termed circulating tumor DNA (ctDNA). The ccfDNA pool is overwhelmingly composed of normal DNA originating from healthy cells [2]. The proportion of ctDNA variants within this pool varies widely based on disease severity [3, 4]. Thus, detection of ctDNA in advanced and/or metastatic disease has been more successful than detection of early-stage or non-metastatic disease [5]. Confounding detection of very low frequency ctDNA variants is noise associated with next-generation sequencing (NGS) [6, 7]. Consequently, ctDNA applications have been largely constrained to detection of known tumor variants [8].

Correction of NGS-related noise has been mainly governed by the assignment of a unique molecular identifier (UMI) to each template DNA molecule prior to library formation [9, 10]. A family is a set of DNA amplicons (PCR duplicates) with the same UMI. Representing a family with a single consensus sequence reduces PCR errors and sequencing artifacts [10, 11]. Although adapters that use a single UMI to track single DNA strands (singleton adapters) reduce noise, the design is vulnerable to early PCR errors (S1 Fig). Subsequent adapter designs labeled double-stranded DNA through integration of dual UMIs to correct early PCR errors with a theoretical background error rate of less than one error per billion nucleotides sequenced [12]. However, poor ligation efficiency resulted in sample loss [13]–an adverse effect particularly problematic in circulating cell-free DNA (ccfDNA) applications where input material is limiting. Recently, dual UMI adapters have been developed with an improved ligation efficiency [14]. Here, we first investigated the effectiveness of error correction during NGS of ccfDNA between single-stranded and double-stranded DNA UMI labeling. The double-stranded DNA adapter design used herein incorporates dual UMIs with dual indexing to concomitantly reduce errors caused by index hopping (duplex adapters, S2 Fig) [15]. Subsequently, we sought to identify and suppress potential sources of residual NGS-related noise to measure the error’s effect on the overall noise profile.

## Results and discussion

We first compared ligation efficiency between the singleton and duplex adapters during library preparation of low-input DNA samples. We found that the overall ligation efficiency of the duplex adapter was higher compared to the singleton adapter (~74% vs. ~58%, respectively; S3 Fig). This observation may be due, at least in part, to the predominantly single-stranded character of the singleton adapter structure (~56 nt unpaired compared to ~19 nt unpaired in the duplex adapter; S2a and S1a Figs, respectively) which may interfere with ligation on account of secondary structure formation, reduced affinity to ligase, and/or increased adapter dimer formation [16]. Regardless, the duplex adapter used in this study was not limited by a reduced ligation efficiency. We further confirmed that the ligation efficiency for duplex adapters remained high (71.7±0.5%) when low-input cancer patient ccfDNA was used (S3 Fig). This observed ligation efficiency is modestly higher than previously reported [14], which may be attributable to differences in ligation protocols (S3 Fig).

**Fig 1:**
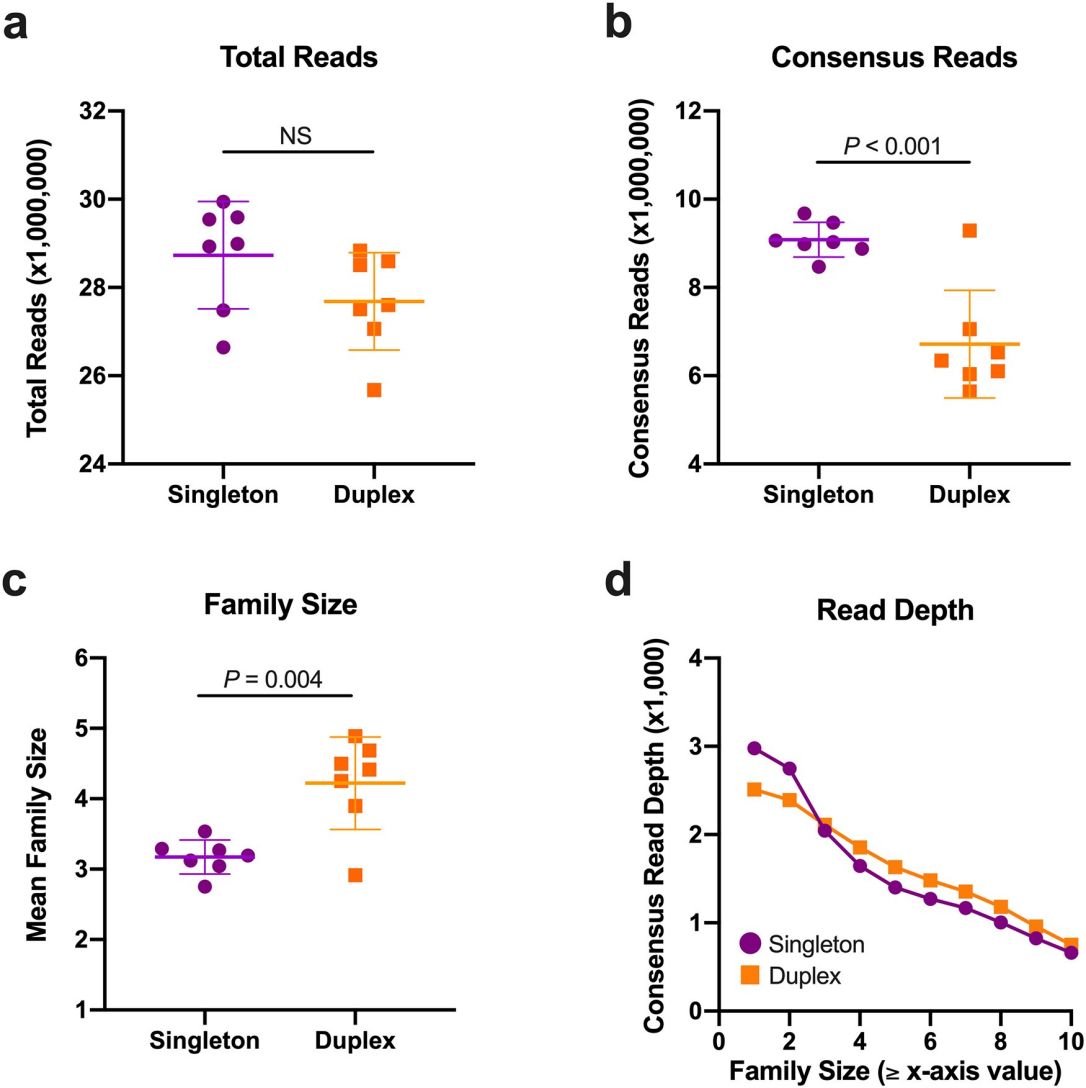
Comparison of sequencing metrics between adapter types. The total number of reads where both read 1 and read 2 were present was similar between the singleton and duplex adapter groups (**a**). However, after consensus sequence determination, there were significantly fewer consensus sequences (**b**) and larger overall family sizes (**c**) in the duplex adapter group. Read depth (**d**) was greater in the duplex adapters at larger family sizes. Bar and whiskers represent mean±SD. Data points shown in (**d**) represent the mean value from the seven control samples.

We then sought to determine the extent of error reduction afforded by the duplex adapters compared to singleton adapters in ccfDNA, where error was defined as the percentage of nonreference alleles (NRAs; GRCh37 reference genome) amongst all consensus reads of exonic bases. Buffy coat DNA and ccfDNA were isolated from seven healthy controls (85.7% female; age range: 28–60 years; median/mean age 39/40.4 years). Two ccfDNA libraries were independently produced for each control using 10 ng of ccfDNA as the initial input–one library with singleton adapters and one library with duplex adapters. Although a similar number of total paired reads resulted from singleton and duplex adapter sequencing (Fig 1a), the duplex adapter group had fewer consensus sequences (Fig 1b) and a 23.1±14.5% larger average family size (Fig 1c), where family size is defined as the minimum number of PCR duplicates that yield a single consensus sequence. Read depth was greater in the singleton adapter group up to family size ≥3 consistent with the generation of larger family sizes by the duplex adapters (Fig 1d). Notably, read depth at family size ≥1 is <3,000X for both adapters (Fig 1d) despite using ~28 million reads per sample (Fig 1a). Assuming lossless procedures during adapter ligation and capture enrichment, the theoretical consensus read depth limit for a 10 ng initial library input is ~2,800X (using an average weight of 650 Da per base pair and a genomic length of 3.3×10^9^ base pairs). The higher than expected read depth reported herein at smaller family sizes is likely attributable to use of exact UMI matching allowing single base pair UMI errors to become unique molecules and falsely elevate read depth. In accord, this effect is reduced at larger family sizes (Fig 1d). Importantly, in the duplex adapter group, we identified both strands from the same initial DNA molecule in only 0.13±0.02% of the sequencing reads. Thus, all results for the duplex adapters are based on consensus data from the initial consensus collapse of each strand (S2 Fig).

**Fig 2:**
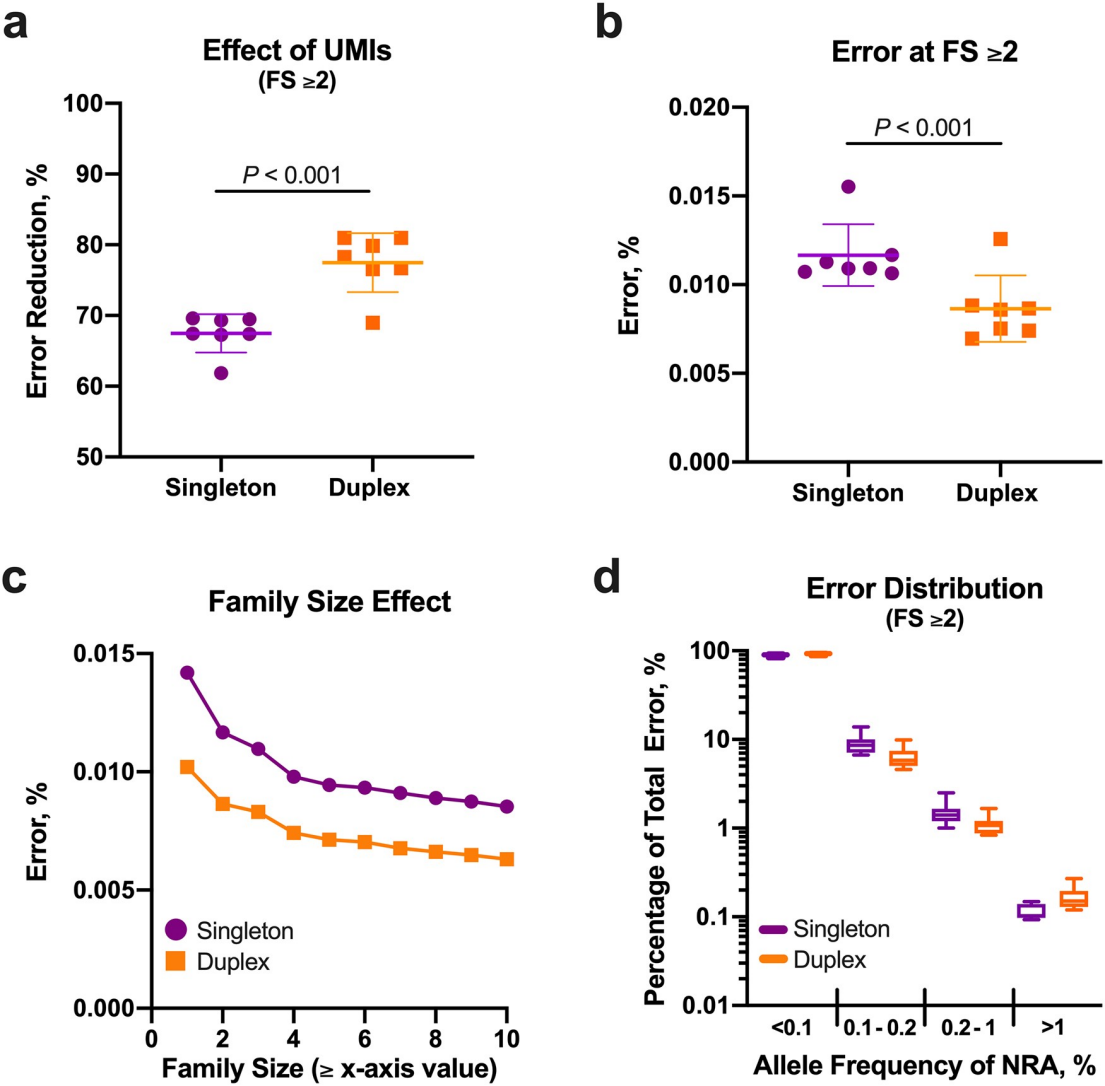
Use of singleton and duplex adapters to reduce noise. Compared to singleton adapters, consensus sequences derived from duplex adapters provided greater error correction (**a**) and lower error (**b**) at family size (FS) ≥2. The gain in error correction using duplex adapters was similar regardless of family size and increments in family size reduced error regardless of adapter type (**c**). Although errors most commonly occurred at an allele frequency <0.1%, a substantial portion of errors had an allele frequency >0.1% (**d**). Bar and whiskers represent mean±SD. Data points shown in (**c**) represent the mean value from the seven control samples.

Error without UMI collapsing was significantly greater using duplex adapters compared to singleton adapters (0.038±0.002 vs. 0.036±0.002%, respectively; *P* = 0.007; S4 Fig), but the difference was relatively small with an increase in the relative error of 7.3±4.9% using the duplex adapters. Index hopping across all duplex adapter groups was measured at <0.02% indicating the occurrence in singleton adapters was unlikely to be a principal source of error because libraries were identically prepared. Compared to the error prior to UMI consensus determination, duplex adapters significantly reduced error more than singleton adapters at family size ≥2 (77.5±4.2 vs. 67.5±2.7% error reduction, respectively; *P* < 0.001; Fig 2a). At family size ≥2, duplex adapters reduced error relative to singleton adapters by only 26.3±5.9% (Fig 2b) and this relative error reduction remained similar regardless of family size (Fig 2c). Approximately 90% of the observed NRAs (i.e., error) occurred with an allele frequency <0.1% (Fig 2d). The mean contribution to total noise of NRAs with an allele frequency between 0.1% and 1% in singleton and duplex adapters was 10.5±2.8% and 7.5±2.1%, respectively (Fig 2d).

**Fig 3:**
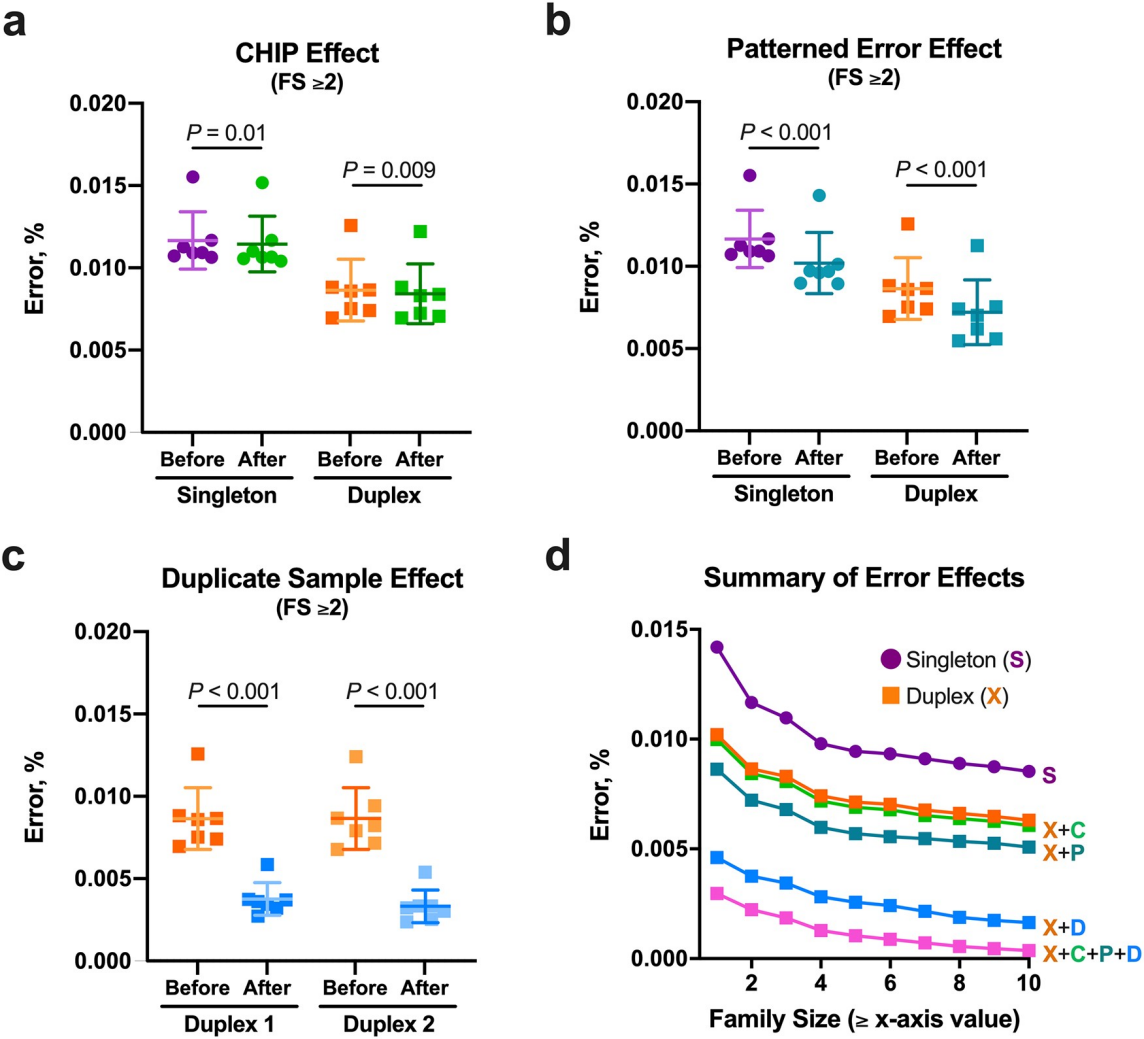
Sources of error and effects of error correction in ccfDNA. Removal of potential CHIP-related artifacts had a relatively small impact on error (**a**), particularly when compared to removal of highly patterned error (i.e., positions with errors in all seven controls; **b**). The greatest reduction in error occurred with application of sample duplicates (**c**). The effects on error from using duplex adapters (X), accounting for CHIP-related artifacts (C), removing positions with highly patterned error (P), and applying data from samples duplicates (D) are shown individually in (**d**). Accounting for all of the different sources of noise yielded the lowest error (**d**, pink), which continually decreased with increments in family size. Data points shown in (**d**) represent the mean value from the seven control samples. FS = family size; CHIP = clonal hematopoiesis of indeterminate potential.

Because there was a persistence of noise in both singleton and duplex adapters even at large family sizes, we explored potential sources of the residual error. NRAs in ccfDNA due to clonal hematopoiesis of indeterminate potential (CHIP) were evaluated by identifying NRAs in buffy coat DNA with an allele frequency between 2% and 30% [17]. We observed, however, that many of these NRAs were present in ≥6 (>85%) buffy coat DNA samples (S5 Fig) suggesting a subset of the potential CHIP-related variants may be attributable to regions difficult to sequence, align, or both (i.e., patterned error). Thus, NRAs present in ≥6 buffy coat DNA samples were removed from the pool of potential CHIP-related variants. Although this cutoff is largely arbitrary, its application allowed us to separately observe the impact on error attributable to potential CHIP-related variants and then subsequently patterned error effects. Removing the potential CHIP-related variants present in 4 of the 7 samples (57%) significantly reduced error, but the reduction in error was <5% at family size ≥2 (Fig 3a). The effect was similar regardless of family size and adapter type (S6 Fig). The modest reduction in overall error associated with CHIP-related artifacts is consistent with previous studies that found CHIP-related variants were uncommon, particularly in individuals younger than 50 years of age [18]. Next, we examined patterned error in ccfDNA regardless of NRA frequency (S7 Fig). Removing positions with NRAs in all seven samples (i.e., highly patterned error) reduced error in the singleton and duplex adapters by 12.8±3.5% (*P* < 0.001) and 17.3±5.0% (*P* < 0.001), respectively (Fig 3b). These common positions accounted for <0.25% of the total exon positions analyzed (S8 Fig). Additional removal of positions with NRAs shared among fewer samples further reduced error (S8a and S8b Fig), but also reduced the total number of positions without error (S8c Fig). However, selecting modestly larger family sizes (e.g., ≥4 or ≥5) mitigated this effect through the elimination of errors occurring at smaller family sizes (S8d and S8e Fig).

Next, we explored the effects of stochastic noise as a source of error. A complete sample duplicate with duplex adapters was generated using a 10 ng input for library preparation from the same seven ccfDNA control samples used previously and following identical procedures. Error was defined as positions with an NRA present in both sample duplicates. Using full library duplicates alone reduced error by 59.4±4.4% (*P* < 0.001) for the duplex adapters at family size ≥2 (Fig 3c). Notably, sequencing the same duplex library twice reduced error by only 19.2±1.3% (*P* < 0.001) at family size ≥2, which was 69.0±3.0% (*P* < 0.001) less error reduction compared to preparation and sequencing of a full library duplicate (S9 Fig). The reduction in error afforded by using the full sample duplicates was greater in magnitude than the combined error correction provided by removal of highly patterned error and correction for potential CHIP-related artifacts. However, we also observed that removing highly patterned error from the duplex full sample duplicate data further reduced error by 40.5±11.3% (*P* < 0.001) at family size ≥2 (S10 Fig). Using duplex adapters, accounting for CHIP artifacts, removing positions with highly patterned error, and including a full sample duplicate reduced error by 94.2±2.5% (*P* < 0.001) at family size ≥2 compared to error prior to UMI consensus determination. Error continued to decline with each family size increment (Fig 3d).

**Fig 4:**
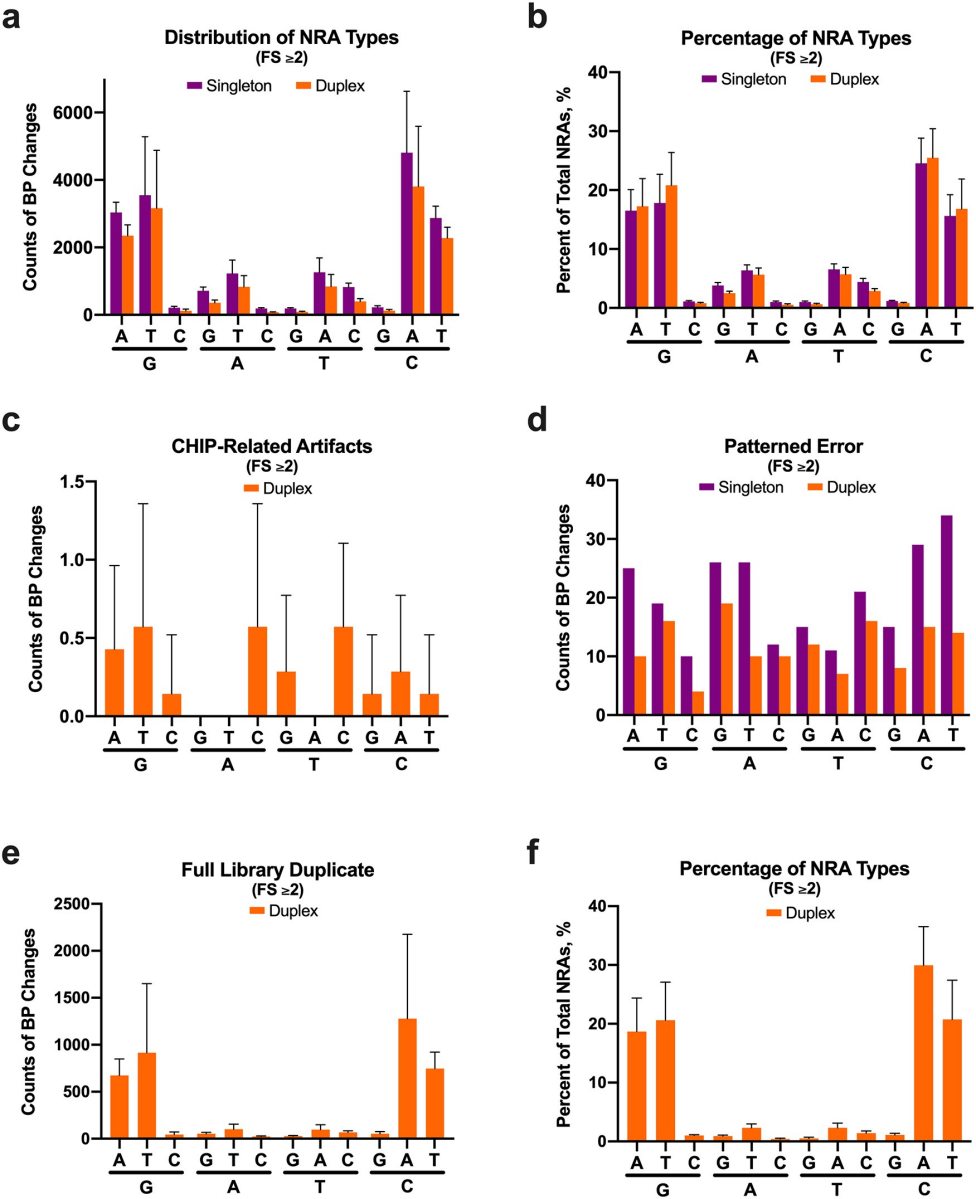
Distribution of NRA types associated with ccfDNA. All data are shown for family size (FS) ≥2. The number of counts for each type of the twelve possible base pair changes is shown in (**a**) for all observed NRAs. In (**b**), the percent of the total NRAs for each base pair change is shown. The distribution for base pair changes associated with CHIP-related artifacts and highly patterned error (NRAs common to all seven samples) is shown in (**c**) and (**d**), respectively. For CHIP-related artifacts, only results from duplex adapters are shown because buffy coat DNA was sequenced only with duplex adapters. In (**d**), error bars are absent because the NRAs were present in all seven samples for each adapter type. In (**e**), the number of counts associated with each type of base pair change present in both of the full library duplex adapter duplicates is depicted. After using full library duplex adapter duplicates to reduce error and removing CHIP-related artifacts and highly patterned error, the distribution of each type of base pair change is shown in (**f**). Error bars represent SD. CHIP = clonal hematopoiesis of indeterminate potential.

Finally, we characterized the base pair changes associated with error in both the singleton and duplex adapter groups. Overall, the most common forms of error (Fig 4a) were two types of transitions (G>A and C>T) and two types of transversions (G>T and C>A) that accounted for >80% of the NRAs identified (Fig 4b). Error was not associated with local GC content (S11 Fig). CHIP-related artifacts (Fig 4c) and patterned error (Fig 4d) did not show a bias towards a particular base pair change. Residual error after using a full library duplicate showed an overall reduction in all types of errors and a proportionately higher reduction in two transitions (A>G and T>C; >83% reduction) and two transversions (A>T and T>A; >87% reduction; Fig 4e). For comparison, the reduction in the other types of errors ranged between 57.1% and 71.3% (Fig 4e). A sequencing duplicate reduced all types of NRAs without affecting the overall pattern (S12 Fig). Using a full library duplicate coupled to removal of CHIP-related artifacts and patterned error altered the original error distribution such that two transitions (G>A and C>T) and two transversions (G>T and C>A) subsequently accounted for 90% of the NRAs (Fig 4f). The error contribution from each of the remaining eight types of possible base pair changes ranged from 0.4% to 2.3% (Fig 4f).

## Conclusions

Collectively, these observations suggest residual error after correction for potential effects of CHIP and patterned error was attributable to very early and random PCR errors during library preparation of low-input ccfDNA. The reduction in noise associated with duplex adapters compared to singleton adapters was likely due to generation of larger family sizes rather than strand pairing as both adapters were susceptible to early PCR errors and we found strand pairing in the duplex adapter group to be uncommon. The stochastic nature of the noise suggests integration of sample duplicates into NGS analytics may be necessary to provide optimal reduction of error [19]. In so doing, the multitude of gene positions provided by panel-capture enrichment may enable untargeted searches of very low frequency ctDNA due to maximum noise suppression, particularly for base pairs changes associated with a low error profile. Although the costs of generating sample duplicate data merits strong consideration within the context of a study design, the reduced costs of sequencing associated with the newer generation of sequencers may mitigate the additional expense.

## Material and methods

### Patient samples and DNA isolation

All procedures were approved by the University of Utah Internal Review Board prior to study initiation (protocol #89989). All participants provided written informed consent. Respondents to flyers posted at the University of Utah from April 2018 to June 2018 seeking healthy volunteers were screened for study participation. Healthy adults (age ≥18 years) without history of cancer, chronic illness, or recent infectious disease were recruited for enrollment. Pregnant women were excluded from the study. Samples were acquired from seven participants (Table 1). The small number of samples used in this study may not be representative of a larger population. Blood samples of study participants were collected in BCT tubes (Streck, La Vista, NE) and processed for buffy coat and plasma extraction within 24 hours. The buffy coat and plasma were separated by centrifugation at 1,900 g x 10 minutes at 4°C and aspirated to new tubes. Plasma was then centrifuged at 16,000 g x 10 minutes at 4°C to remove any cellular debris. The plasma supernatant and the buffy coat were stored at -80°C. White blood cell (WBC) DNA was isolated from the buffy coat using the QIAamp DNA Blood Mini Kit (Qiagen, Germantown, MD) and eluted in a final volume of 100 μL 10 mM Tris-Cl and 0.5 mM EDTA (pH 8.0). Cell-free DNA was isolated from 7–14 mL of plasma using the QIAamp Circulating Nucleic Acid Kit (Qiagen) and eluted in a final volume of 40 μL 10 mM Tris (pH 8.0) and 0.1 mM EDTA.

**Table 1:** Participant demographics.

	Mean±SD or Number (%)
Age	40.4±11.6 yrs
Female	6 (85.7)
Race:	
White, Non-Hispanic	6 (85.7)
White, Hispanic	1 (14.3)

SD = standard deviation

### Preparation of synthetic ligation substrates for ligation efficiency assays

A synthetic gBlock was synthesized by Integrated DNA Technologies (IDT; Coralville, IA; S1 Table). A 165 bp PCR product was generated from lambda DNA by PCR using primer sequences provided in S1 Table.

### DNA input

In assays to determine ligation efficiency 20 ng of DNA input were used. Healthy control libraries were prepared from 10 ng ccfDNA or 100 ng of WBC genomic DNA. WBC genomic DNA was sheared using a focused ultrasonicator (S220, Covaris, Woburn, MA) with a targeted size of 175 bp.

### Library preparation

Template DNA underwent end-repair and A-tailing followed by ligation of adapters (5 μL at 15 μM concentration added to each reaction) using the NEBNext Ultra II DNA Library Prep Kit (New England Biolabs, Ipswich, MA) following the manufacturer’s instructions. For comparison studies shown in S3c Fig, duplicate ccfDNA libraries were prepared using the Kapa Hyper Prep Kit (Roche, Indianapolis, IN). Following the ligation reaction, gBlock DNA and lambda DNA samples underwent SPRI bead cleanup (Agencourt AMPure XP, Beckman Coulter, Indianapolis, IN) using a 2X SPRI ratio and elution volume of 25 μL IDTE. WBC DNA and ccfDNA underwent SPRI bead cleanup using a 1X SPRI ratio and elution volume of 20 μL IDTE followed by 10 cycles of PCR amplification for ccfDNA using the KAPA Library Amplification Kit (Roche, Indianapolis, IN) and following the manufacturer’s instructions. Samples with singleton and duplex adapters were amplified using 5 μL of 20 μM P5/P7 primers and 20 μM indexing primers, respectively. Singleton adapters, duplex adapters, and the associated primers were obtained from IDT.

### Determination of ligation efficiency by densitometry and validation with ddPCR

Ligation efficiency for gBlock, lambda DNA PCR product, and patient ccfDNA input was measured by densitometry. After the ligation step and SPRI bead clean up as described above, 2 μL of the eluate were analyzed using a High Sensitivity D1000 Screentape on a 2200 TapeStation System (Agilent, Santa Clara, CA; S13 and S14 Figs). Free adapter and input DNA were included in separate lanes as migration references. Ligation efficiency was determined by defining regions of unligated, single-end ligated, and dual-end ligated densitometry peaks and quantifying region molarity from reported region concentration and average fragment size (S13 and S14 Figs). Ligation efficiency was defined as percent dual-end ligated product among all ligation products. Ligation efficiencies stated in the main text are the mean±SD of all ligation efficiency measurements by densitometry.

In ligation experiments that used the synthetic gBlock as template, ligation efficiency was orthogonally determined by droplet-digital PCR (ddPCR). Samples were taken following the ligation after the 2X SPRI cleanup and following six cycles of library PCR amplification (S15a Fig). Samples were diluted (10^5^ to 10^7^-fold) prior to ddPCR to obtain copy numbers in the dynamic range of the ddPCR instrument. ddPCR reactions were performed on the QX200 AutoDG ddPCR system (Bio-Rad Laboratories, Hercules, CA). Two ddPCR reactions were set up for each sample, containing either internal primers and the gBlock-specific probe or flanking primers mapping to adapter regions and the gBlock-specific probe (S15b Fig; primer and probe sequences and ddPCR conditions are provided in S1 and S2 Tables). Ligation efficiency by ddPCR was calculated as the fraction of dual-end ligated copy number (flanking primers) among total copy number (internal primers).

The ligation efficiency of gBlock DNA as measured by both ddPCR and densitometry was similar indicating that densitometry was a valid method for analyzing ligation efficiency (S15f Fig). Densitometry results are subsequently reported as measures for ligation efficiency of both gBlock DNA (blunt-ended DNA) and lambda DNA (A-tailed DNA) in S3 Fig.

### Sequencing, alignment, and consensus calling

Buffy coat DNA and ccfDNA libraries underwent panel capture enrichment using a custom designed IDT Xgen capture probe set (118 genes, 124 kb; S3 Table; IDT) followed by paired-end sequencing (2x125 bp) on a HiSeq 2500 (Illumina, San Diego, DA). An identical number of samples were loaded per lane to provide a similar number of reads for each sample and adapter type (S16 Fig). Reads in fastq files were aligned to the GRCh37 reference genome. Singleton and duplex indexed libraries were demultiplexed into individual samples using Illumina’s bcl2fastq application (https://support.illumina.com/sequencing/sequencing_software/bcl2fastq-conversion-software.html).

To generate uncorrected nonconsensus alignments, paired end fastq datasets were aligned to GRCh37 using a standard BWA mem, Picard MarkDuplicate, and GATK polishing snakemake workflow (https://github.com/HuntsmanCancerInstitute/Workflows/blob/master/Alignment/alignQC_1.3.svgsee also alignQC_1.3.sm and alignQC_1.3.sh). To generate error corrected consensus alignments, a UMI aware workflow was developed (https://github.com/HuntsmanCancerInstitute/Workflows/blob/master/Alignment/MolBarcodes/consensusAlignQC_0.4.svg see also consensusAlignQC_0.4.sm and consensusAlignQC_0.4.sh). This makes use of a variety of USeq tools (https://github.com/HuntsmanCancerInstitute/USeq) to identify alignment pairs with the same unclipped start position and group those with identical (i.e., 100% barcode similarity) 8-mer (singleton adapter) or 6-mer (duplex adapter) UMIs into families. Read sequence is extracted from each alignment in the family and a consensus sequence called by examining each base position in the sequence stack. Those with >0.66 concordance were assigned the predominant base and the maximum observed quality score, otherwise, an N base is assigned with zero quality. Consensus paired end reads are realigned and inserted into the original alignment file in place of the corresponding family members.

### Error analysis

Error analysis was restricted to exons (S17 Fig). The USeq EstimateErrorRates application calculates base level error rates observed in quality alignments (≥ MQ20) from normal germline sequencing datasets where an error is any single nucleotide variant that does not match the reference genome (GRCh37; i.e., nonreference allele, NRA). The USeq EstimateErrorRates parses a Samtools mpileup alignment stack for regions of 7 adjacent bases with adequate read depth (≥100, Q20 bases), no observed indels, and no indication of heterozygous or homozygous single nucleotide variants (allele frequencies ≤0.4). Good quality (≥Q20), nonreference, center base observations in each passing region are tabulated. These are used to calculate error rates for each exonic base as well as the total error observed from quality alignments and quality bps. GC content was calculated from the ±10 bp of the NRA position.

The USeq MpileupParser works in a similar fashion by parsing a Samtools mpileup alignment stack. It identifies genomic base positions that contain a minimum aligned base depth of 100. Only quality alignments (≥MQ20) and quality bases (≥Q20) are counted. Positions with evidence of a heterozygous or homozygous allele (allele frequency > 0.4) are ignored. It outputs a bed file of each passing base with its observed nonreference allele frequency. This can be used to identify regions with high error rates.

### Calculation of index hopping error rates

After sequencing, samples were demultiplexed with unique dual indexing (UDI). UDI associates each samples’ reads with two distinct 8 base barcodes positioned on opposite ends of a DNA insert, which Illumina refers to as index 1 (i7) and index 2 (i5). Reads with correct UDI pairing (i7, i5) are unambiguously mapped to a sample. Additionally, UDI enables quantification of ambiguous reads exhibiting ‘index hopping’. Reads with index hopping contain mixed-sample barcodes in their UDI pairing. For illustration, suppose reads from sample A has UDI pair (A-i7, A-i5) and reads from sample B has UDI pair (B-i7, B-i5). Then ambiguous reads with mixed sample UDI pairs of the form (A-i7, B-i5) or (B-i7, A-i5), are instances of index hopping.

The index hopping rate was calculated by including an exhaustive list of mixed-sample UDI pairs into the configuration file required by Illumina’s demultiplexing software, bcl2fastq (v2.20.0.422). The software counts the number of reads associated with each specified UDI pair, allowing for up to 1 base mismatch. The rate of index hopping is defined to be the sum total of reads mapping unambiguously to mixed-sample UDI pairs and then divided by the total number of reads in a sequencing lane. For completeness, the demultiplexing software simultaneously calculated the rate of sample-associated reads (i.e., correct UDI pairs are also included in the configuration file) as well as the rate of reads unaffiliated with a sample for reasons besides index hopping.

### Statistics

Reported values (X±Y) represent the mean (X) and standard deviation (Y) for the seven samples. For paired samples, the paired t-test was used. Repeated measures ANOVA with a Greenhouse-Geisser correction determined differences within groups. Post hoc tests using Bonferroni correction was applied for comparisons between pairs of samples. The independent t-test was used for comparison of two independent samples and Levene’s test for inequality determined equal or unequal variance. The one-sample t-test was applied for comparisons of percent change to zero. Bars on data plots identify the mean value, while whiskers identify standard deviation. All statistical analysis was performed in SPSS (Version 25, IBM). Statistical significance was defined as *P* < 0.05.

## Supporting information

S1 Table Sequences of synthetic oligonucleotides, primers, and ddPCR probe. (PDF)

S2 Table ddPCR thermocycling conditions. (PDF)

S3 Table 118 genes included in next-generation capture panel. (PDF)

S1 Fig Schematic for singleton adapters. In (**a**), the sequence for the complete unligated singleton adapter is shown. Both the single index (i7) and the single unique molecular identifier (UMI) are 8 bp in length. The ‘T*C’ denotes a phosphorothioate bond. The sequences for the P7 and P5 primers are also shown along with their colored matched segments in the adapter. In (**b**), the template DNA associated with the primer represents either the primer recognition site (darker coloring) or the primer sequence (lighter coloring). During the first cycle of PCR, only the P7 primer is used for amplification, which yields two amplicons with separate UMIs. Note that the P5 primer recognition site is generated during the first cycle of PCR allowing for both P7 and P5 primers to be used in subsequent PCR cycles. Because two amplicons with separate UMIs are produced on the first PCR cycle, two separate families of PCR amplicons are generated and independently used for consensus sequence interpretation. A true variant (purple dots) is shown to amplify consistently in both families. However, the introduction of a PCR error (red dots) during the first cycle of PCR becomes isolated to only one of the families. During subsequent PCR cycles, if the template with the error is selectively propagated more than the template without the error, the error can become overrepresented and generate a false positive during consensus calling (left side of 3^rd^ PCR cycle).
(PDF)

S2 Fig Schematic for duplex adapters. In (**a**), the sequence for the complete unligated duplex adapter is shown. The dual UMIs (3 bp) are embedded within the short double-stranded segment of the adapter, while the single stranded segments consist of the duplex primer locations (D7 and D5). The ‘T*G’ denotes a phosphorothioate bond. The duplex primers (i7 indexing primers, i5 indexing primer) are shown with colored matched sequences in the adapter. The primers contain the indices and the P7 and P5 primer recognition sites, which have been colored to match the sequences shown in S1 Fig. In (**b**), the template DNA associated with the primer represents either the primer recognition site (darker coloring) or the primer sequence (lighter coloring). During the first cycle of PCR, only the D7 primer is used for amplification, which yields two amplicons both harboring the same two UMIs, a single index, and the P7 primer sequence (lighter color). Because the second index and the P5 primer sequence has not been added yet, these are referred to as ‘partial products.’ The D5 recognition site is generated during the first cycle of PCR allowing for both D7 and D5 primers to be used in subsequent PCR cycles. Amplification with the D5 primer adds the second index and the P5 primer sequence (lighter color). With each subsequent PCR cycle, both a ‘partial product’ and a ‘full product’ are generated. The partial product results from amplification of previous partial products with the D7 primer. The full product is derived from amplification of a partial product with the D5 primer or a full product with either the D7 or D5 primer. Consensus sequence determination is a two-step process. First (Step 1), all aligned molecules with the same UMI are collapsed into a single sequence. During this initial step, molecules from the two original strands are not combined because the order of the UMI at the 3mer level from different strands is different even though the bases are the same. Specifically, if the UMI for strand A is ‘abc-def’ then the UMI for strand B is ‘def-abc.’ Next (Step 2), the UMI ordering is used to identify aligned paired strands that then undergo a second consensus sequence determination. Theoretically, the use of paired strand information enables removal of early PCR errors. As before, a true variant (purple dots) is shown to amplify consistently. The introduction of a PCR error (red dots) during the first cycle of PCR does not generate a false positive in a consensus sequence compared to the singleton adapters because of the second consensus sequence determination. However, duplex adapters are vulnerable to false positives from PCR errors if paired strand sequence data is infrequent. In this study, paired strand sequence data was present for <0.2% of consensus reads. Thus, all results for the duplex adapters are based on the Step1 consensus sequences.
(PDF)

S3 Fig Ligation efficiency. For both blunt-ended DNA (**a**) and A-tailed DNA (**b**) the ligation efficiency was significantly greater with the duplex adapters than the singleton adapters. On low-input ccfDNA from patients with pancreatic ductal adenocarcinoma (**c**), a significant difference between ligation protocols was observed. For all reported findings in this study, protocol 1 was used. Procedures associated with protocol 2 were identical except as indicated by the manufacturer’s instructions specific to the different ligation kit that was tested.
(PDF)

S4 Fig Error prior to use of UMIs. The error prior to using UMIs for generation of consensus sequences was higher for the duplex adapters compared to the singleton adapters. Bar and whiskers represent mean±SD.
(PDF)

S5 Fig Potential CHIP-related artifacts. Nonreference alleles (NRAs) in buffy coat DNA (family size ≥2) with an allele frequency between 2% and 30% were identified in each sample and then graphed based on allele frequency and occurrence in other buffy coat DNA samples. Potential CHIP-related artifacts were present in four out of seven samples. The allele frequencies for each potential CHIP-related variant associated with each patient is shown (triangles, circles, and squares–similar symbols are from the same sample). If present in more than one sample, the NRA frequency is displayed from a single sample.
(PDF)

S6 Fig Effect of removing noise due to CHIP artifacts from singleton and duplex adapters at different family sizes. Note the contribution of CHIP artifacts to noise is relatively small in these control samples. Data points represent the mean value from the seven control samples.
(PDF)

S7 Fig Patterned error in ccfDNA. Nonreference alleles (NRAs) in ccfDNA (family size ≥2) were identified in each sample and then graphed based on allele frequency and occurrence in other ccfDNA samples for both singleton (**a**) and duplex (**b**) adapters. The distribution of allele frequencies is from a single sample (the gray triangles correspond to the sample represented with gray triangles in S5 Fig).
(PDF)

S8 Fig Effects of patterned error on noise level and footprint size. In (**a**) and (**b**), the original error rate for different family sizes is shown as the top line for singleton and duplex adapters, respectively. Each subsequent line represents the reduction in noise due to removing locations with patterned error. As the sequential lines lighten in color, relatedness is reduced. For example, the first line indicates the error rate when locations are removed that have error in all seven samples. The next line indicates the error rate when locations are removed with error in at least six of the samples and so forth. The bottom line represents the error rate when locations are removed when error is present at a location in two or more samples. The rise in error seen at the lower degrees of relatedness at increment family sizes is due to the greater effects of noise elimination through consensus sequence determination rather than patterned error removal. Although the error rate is progressively reduced, note the effect on the panel footprint at family size ≥2 (**c**). The more lenient criteria used to define patterned error results in a progressively reduced panel footprint leaving fewer positions available for subsequent analysis. However, in both singleton (**d**) and duplex (**e**) adapters the reduction in panel size associated with using reduced relatedness can be mitigated by using larger family sizes. Data points in all figure elements represent the mean value from the seven control samples.
(PDF)

S9 Fig Effect of a sequencing duplicate compared to a full sample duplicate on error. All data shown are from duplex adapters. ‘Before’ represents the NGS error rate in ccfDNA from the second full library generated with duplex adapters (orange squares). ‘Seq’ represents the error rate associated with sequencing the same capture-enriched library twice. ‘Full’ represents the error rate associated with generation of a full sample duplicate through an independent library formation (the values are the same as shown in Fig 3c, Duplex 2). For both ‘Seq’ and ‘Full,’ error is defined as the same NRA occurring in both corresponding duplicates. Note the substantial reduction in error associated with production of a full sample duplicate compared to sequencing the same library twice. This observation is consistent with early and random PCR errors during library formation being a principal source of noise in NGS.
(PDF)

S10 Fig Effect of removing additional sources of error in duplicate sample data. The initial error after using sample duplicate data (D) is shown for each duplex adapter group at family size (FS) ≥2. Sources of noise, singularly and in combination, were then removed from the duplicate sample data to determine effects on error. Overall, the mean values were significantly different within each sample duplicate–Duplex 1 (*F*(1.635,9.810) = 146.252, *P* < 0.001) and Duplex 2 (*F*(1.381.8.287) = 122.815, *P* < 0.001). Statistically significant differences between each group within each duplicate are indicated in the figure. Removing CHIP artifacts (+C) had a minor effect. In contrast, removing patterned error (+P) substantially reduced the overall error associated with duplex sample duplicates. The error rate reduction associated with accounting for both CHIP artifacts and patterned error (+C, +P) was largely due to the patterned error contribution.
(PDF)

S11 Fig Local GC content associated with each type of NRA. FS = family size.
(PDF)

S12 Fig Distribution of NRA types present in both sequencing duplicates. FS = family size.
(PDF)

S13 Fig Densitometry analysis of singleton adapter ligation. In (**a**), a schematic of the insert (red) and adapter is shown. On densitometry, the adapter migrated at 288 bp (**b**), a substantial shift from the expected length. This difference is most likely due to the ~56 nt unpaired single-stranded segments which alter the electrophoretic mobility of the adapter compared to double-stranded DNA of a similar length. For example, the peak of the165 bp double-stranded DNA input occurred at the expected size (**c**). Using (**b**) and (**c**) as references, the unligated, single-end, and dual-end ligation products can be identified (**d**). After PCR (**e**), the fully double-stranded ligated product (**f**) occurred at a size consistent with a 165 bp insert and dual-end adapters.
(PDF)

S14 Fig Densitometry analysis of duplex adapter ligation. In (**a**), a schematic of the insert (red) and adapter is shown. On densitometry, the adapter migrated at 75 bp due to the presence of single-stranded regions (**b**). Because the single-stranded segments were significantly shorter compared to the singleton adapters, the observed electrophoretic mobility shift was less pronounced for duplex adapters (compare with S10b Fig). The peak of the double-stranded 165 bp DNA input occurred at the expected size (**c**). Using (**b**) and (**c**) as references, the unligated, single-end, and dual-end ligation products can be identified (**d**). After PCR (**e**), the fully double-stranded ligated product (**f**) occurred at a size consistent with a 165 bp insert and dual-end adapters.
(PDF)

S15 Fig ddPCR for validating measurements of ligation efficiency with densitometry. The diagram in (**a**) illustrates the steps in library preparation for adapter ligation. For ddPCR quantitative analysis, a sample was taken after ligation cleanup and after PCR cleanup. For densitometry quantitative analysis (TapeStation), a sample was taken after ligation cleanup. To determine ligation efficiency using ddPCR, two separate reactions were performed. One reaction included a probe for *EGFR* and a primer pair flanking the probe (internal, **b**). The second reaction included the same *EGFR* probe and a primer pair on the adapters flanking the insert (flanking, **b**). Thus, ligation efficiency was based on the ratio of absolute copy number counts from the flanking primer/probe set (i.e., dual-end ligated) to the internal primer/probe set (i.e., the reference DNA copy number). Ligation efficiency by ddPCR was calculated after ligation cleanup (**c**). ddPCR measurements after PCR amplification and suppression/removal of any unligated adapters and DNA inserts (**d**) were performed to show a similar amount of signal from both internal and external primers as indication that the measured ligation efficiency was not principally attributable to differences in PCR efficiency between the internal and external primer pairs. Ligation efficiency by densitometry was done with Tapestation analysis and following ligation clean-up (**e**). The method for measuring unligated, single-end, and dual-end products is described in S13 and S14 Figs for singleton and duplex adapters, respectively. The ligation efficiency measured by densitometry was similar to that measured by ddPCR (**f**).
(PDF)

S16 Fig FASTQ reads. The total number of FASTQ reads was similar between singleton and duplex adapters.
(PDF)

S17 Fig Panel size based on exon coverage relative to family size. For family size <5, the panel size is ~101 kb for both the duplex and singleton adapters. Note that the panel size decays rapidly for family size ≥5 as fewer exon positions have consensus reads at larger family sizes. Data points represent the mean value from the seven control samples.
(PDF)
