# Supplementary material for: The stochastic nature of errors in next-generation sequencing of circulating cell-free DNA
Source: PLoS One. 2020 Feb 21;15(2):e0229063. doi: 10.1371/journal.pone.0229063 (PMC7034809; doi:10.1371/journal.pone.0229063)
Supplement: S1 Table — (PDF) [file pone.0229063.s001.pdf]

Table S1: Sequences of synthetic oligonucleotides, primers, and ddPCR probe

**165bp gBlock sequence (5' – 3')**	
GTGGACAACCCCCACGTGTCGGATCGCAGGTGAAATGCCGCCTGCTGGGCATCTGCCTCACCTCCACCGTGCAGCTCATCATGCAGCTCATGCCCTTCGGCTGCCTCCTGGACTATGTCCGGGAACTGCCAGTATTCTCGACGGACAAAGACAATATTGGCTCCC	
**Primer sequences (5' – 3')**	
gBlock Internal FWD	CCTCACCTCCACCGTGCA
gBlock Internal REV	CCTCACCTCCACCGTGCA
Duplex Flanking FWD	TCTTTCCCTACACGACGC
Duplex Flanking REV	GTGACTGGAGTTCAGACGTG
Singleton Flanking FWD	AATGATACGGCGACCACCGA
Singleton Flanking REV	GTGACTGGAGTTCAGACGTGTGC
165 bp Lambda FWD	TGAACTGATTGCCCGTCTCC
165 bp Lambda REV	ACATTTTCCCGGCTGAGAGG
**ddPCR hydrolysis probe sequence**	
/56-FAM/T+CATC+A+T+GC/ZEN/A+GC+TC/3IABkFQ/	
