# Supplementary material for: The stochastic nature of errors in next-generation sequencing of circulating cell-free DNA
Source: PLoS One. 2020 Feb 21;15(2):e0229063. doi: 10.1371/journal.pone.0229063 (PMC7034809; doi:10.1371/journal.pone.0229063)
Supplement: S2 Table — (PDF) [file pone.0229063.s002.pdf]

**Table S2: ddPCR thermocycling conditions**

| | |
|---|---|
| **1 cycle** | |
| 10 min | 95°C |
| **45 cycles** | |
| 15 sec | 94°C |
| 15 sec | 58°C |
| 45 sec | 60°C |
| **1 cycle** | |
| 10 min | 98°C |
| **hold** | 12°C |
