# Supplementary material for: The stochastic nature of errors in next-generation sequencing of circulating cell-free DNA
Source: PLoS One. 2020 Feb 21;15(2):e0229063. doi: 10.1371/journal.pone.0229063 (PMC7034809; doi:10.1371/journal.pone.0229063)
Supplement: S3 Table — (PDF) [file pone.0229063.s003.pdf]

Table S3. 118 genes included in next-generation capture panel.

ABCB1	*ABCC9*	*ABL1*	*ADAM29*	*AFM*	*AIFM3*	*AKT1*
ALK	*ANKRD36*	*APC*	*ATM*	*ATRX*	*BRAF*	*CALCR*
CARD6	*CDH1*	*CDH18*	*CDH9*	*CDHR3*	*CDKN2A*	*CDX4*
CIC	*COL1A2*	*CTNNB1*	*CXorf22*	*DCAF12L2*	*DDR2*	*DRD5*
DYNC1I1	*EGFR*	*ERBB2*	*ERBB4*	*ERCC1*	*FBXW7*	*FGA*
FGFR1	*FGFR2*	*FGFR3*	*FIP1L1*	*FLT3*	*FOXR2*	*FRMD7*
FUBP1	*GABRA1*	*GABRA6*	*GABRB2*	*GCSAML*	*GNA11*	*GNAQ*
GNAS	*GPX5*	*H3F3AP4*	*HIST1H3B*	*HRAS*	*IDH1*	*IDH2*
IL18RAP	*JAK2*	*JAK3*	*KDR*	*KEL*	*KIT*	*KLF4*
KRAS	*KRTAP20-2*	*LCE4A*	*LRRC55*	*LUM*	*LZTR1*	*MAP2K1*
MET	*MMP13*	*MROH2B*	*MSH6*	*MTOR*	*NF1*	*NF2*
NLRP5	*NOTCH1*	*NOVA1*	*NRAS*	*ODF4*	*PARD6B*	*PDGFRA*
PIK3CA	*PIK3R1*	*PLCH2*	*PODNL2*	*PTEN*	*QKI*	*RB1*
RET	*RFX6*	*RPL5*	*SCN9A*	*SEMA3C*	*SEMA3E*	*SIGLEC8*
SLC26A3	*SMAD4*	*SMO*	*SPTA1*	*STAG2*	*STK11*	*SULT1B1*
TCHH	*TERT*	*TMEM147*	*TP53*	*TPTE2*	*TRAF7*	*TRIM51*
TRPV6	*UGT2A3*	*VHL*	*WNT2*	*ZNF844*	*ZNF99*	
