# Supplementary material for: The stochastic nature of errors in next-generation sequencing of circulating cell-free DNA
Source: PLoS One. 2020 Feb 21;15(2):e0229063. doi: 10.1371/journal.pone.0229063 (PMC7034809; doi:10.1371/journal.pone.0229063)
Supplement: S1 Fig — In (a), the sequence for the complete unligated singleton adapter is shown. Both the single index (i7) and the single unique molecular identifier (UMI) are 8 bp in length. The ‘T*C’ denotes a phosphorothioate bond. The sequences for the P7 and P5 primers are also shown along with their colored matched segments in the adapter. In (b), the template DNA associated with the primer represents either the primer recognition site (darker coloring) or the primer sequence (lighter coloring). During the first cycle of PCR, only the P7 primer is used for amplification, which yields two amplicons with separate UMIs. Note that the P5 primer recognition site is generated during the first cycle of PCR allowing for both P7 and P5 primers to be used in subsequent PCR cycles. Because two amplicons with separate UMIs are produced on the first PCR cycle, two separate families of PCR amplicons are generated and independently used for consensus sequence interpretation. A true variant (purple dots) is shown to amplify consistently in both families. However, the introduction of a PCR error (red dots) during the first cycle of PCR becomes isolated to only one of the families. During subsequent PCR cycles, if the template with the error is selectively propagated more than the template without the error, the error can become overrepresented and generate a false positive during consensus calling (left side of 3rd PCR cycle). (PDF) [file pone.0229063.s004.pdf]

a

Annealed singleton adapter:

```
5’-Phos-GATCGGAAGAGCACACGTCTGAACTCCAGTCAC[i7]ATCTCGTATGCCGTCTTCTGCTTG
            ||||||||||||
        T*CTAGCCTTCGCACTCGCAATCCCTTTCTCACA[NNNNNNNN]ACATCTAGAGCCACCAGCGGCATAGTAA-5’
```

P7 primer: 5’-CAAGCAGAAGACGGCATACGA

P5 primer: 5’-AATGATACGGCGACCACCGA

b

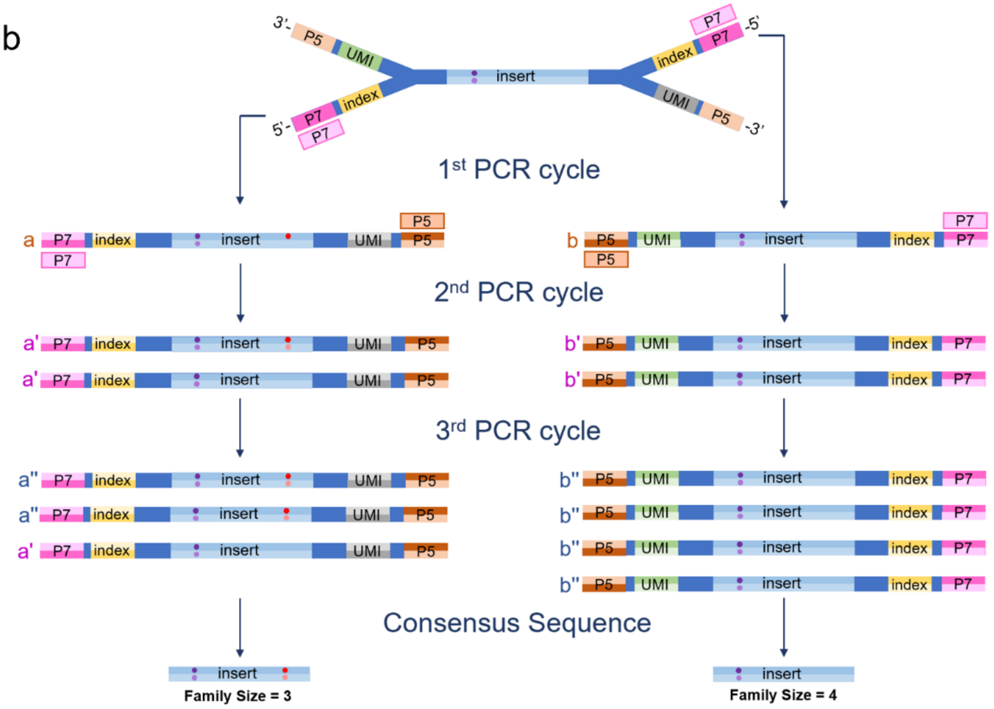

S1 Fig. Schematic for singleton adapters. In (**a**), the sequence for the complete unligated singleton adapter is shown. Both the single index (i7) and the single unique molecular identifier (UMI) are 8 bp in length. The ‘T*C’ denotes a phosphorothioate bond. The sequences for the P7 and P5 primers are also shown along with their colored matched segments in the adapter. In (**b**), the template DNA associated with the primer represents either the primer recognition site (darker coloring) or the primer sequence (lighter coloring). During the first cycle of PCR, only the P7 primer is used for amplification, which yields two amplicons with separate UMIs. Note that the P5 primer recognition site is generated during the first cycle of PCR allowing for both P7 and P5 primers to be used in subsequent PCR cycles. Because two amplicons with separate UMIs are produced on the first PCR cycle, two separate families of PCR amplicons are generated and independently used for consensus sequence interpretation. A true variant (purple dots) is shown to amplify consistently in both families. However, the introduction of a PCR error (red dots) during the first cycle of PCR becomes isolated to only one of the families. During subsequent PCR cycles, if the template with the error is selectively propagated more than the template without the error, the error can become overrepresented and generate a false positive during consensus calling (left side of 3rd PCR cycle).
