# Supplementary material for: The stochastic nature of errors in next-generation sequencing of circulating cell-free DNA
Source: PLoS One. 2020 Feb 21;15(2):e0229063. doi: 10.1371/journal.pone.0229063 (PMC7034809; doi:10.1371/journal.pone.0229063)
Supplement: S2 Fig — In (a), the sequence for the complete unligated duplex adapter is shown. The dual UMIs (3 bp) are embedded within the short double-stranded segment of the adapter, while the single stranded segments consist of the duplex primer locations (D7 and D5). The ‘T*G’ denotes a phosphorothioate bond. The duplex primers (i7 indexing primers, i5 indexing primer) are shown with colored matched sequences in the adapter. The primers contain the indices and the P7 and P5 primer recognition sites, which have been colored to match the sequences shown in S1 Fig. In (b), the template DNA associated with the primer represents either the primer recognition site (darker coloring) or the primer sequence (lighter coloring). During the first cycle of PCR, only the D7 primer is used for amplification, which yields two amplicons both harboring the same two UMIs, a single index, and the P7 primer sequence (lighter color). Because the second index and the P5 primer sequence has not been added yet, these are referred to as ‘partial products.’ The D5 recognition site is generated during the first cycle of PCR allowing for both D7 and D5 primers to be used in subsequent PCR cycles. Amplification with the D5 primer adds the second index and the P5 primer sequence (lighter color). With each subsequent PCR cycle, both a ‘partial product’ and a ‘full product’ are generated. The partial product results from amplification of previous partial products with the D7 primer. The full product is derived from amplification of a partial product with the D5 primer or a full product with either the D7 or D5 primer. Consensus sequence determination is a two-step process. First (Step 1), all aligned molecules with the same UMI are collapsed into a single sequence. During this initial step, molecules from the two original strands are not combined because the order of the UMI at the 3mer level from different strands is different even though the bases are the same. Specifically, if the UMI for strand A is ‘abc-def’ t [file pone.0229063.s005.pdf]

a Annealed duplex adapter:

```
5'-Phos-C[NNN]AGATCGGAAGAGCACACGTCTGAACTCCAGTC-3'
        |  |||  |||||||||||||
    3'-T*G[nnn]TCTAGCCTTCTCGCAGCACATCCCTTTCTCACA-5'
```

i7 indexing primer: 5'-CAAGCAGAAGACGGCATACGAGAT[i7]GTGACTGGAGTTCAGACGTGT-3'

i5 indexing primer: 5'-AATGATACGGCGACCACCGAGATCTACAC[i5]ACACTCTTTCCCTACACGAC-3'

b

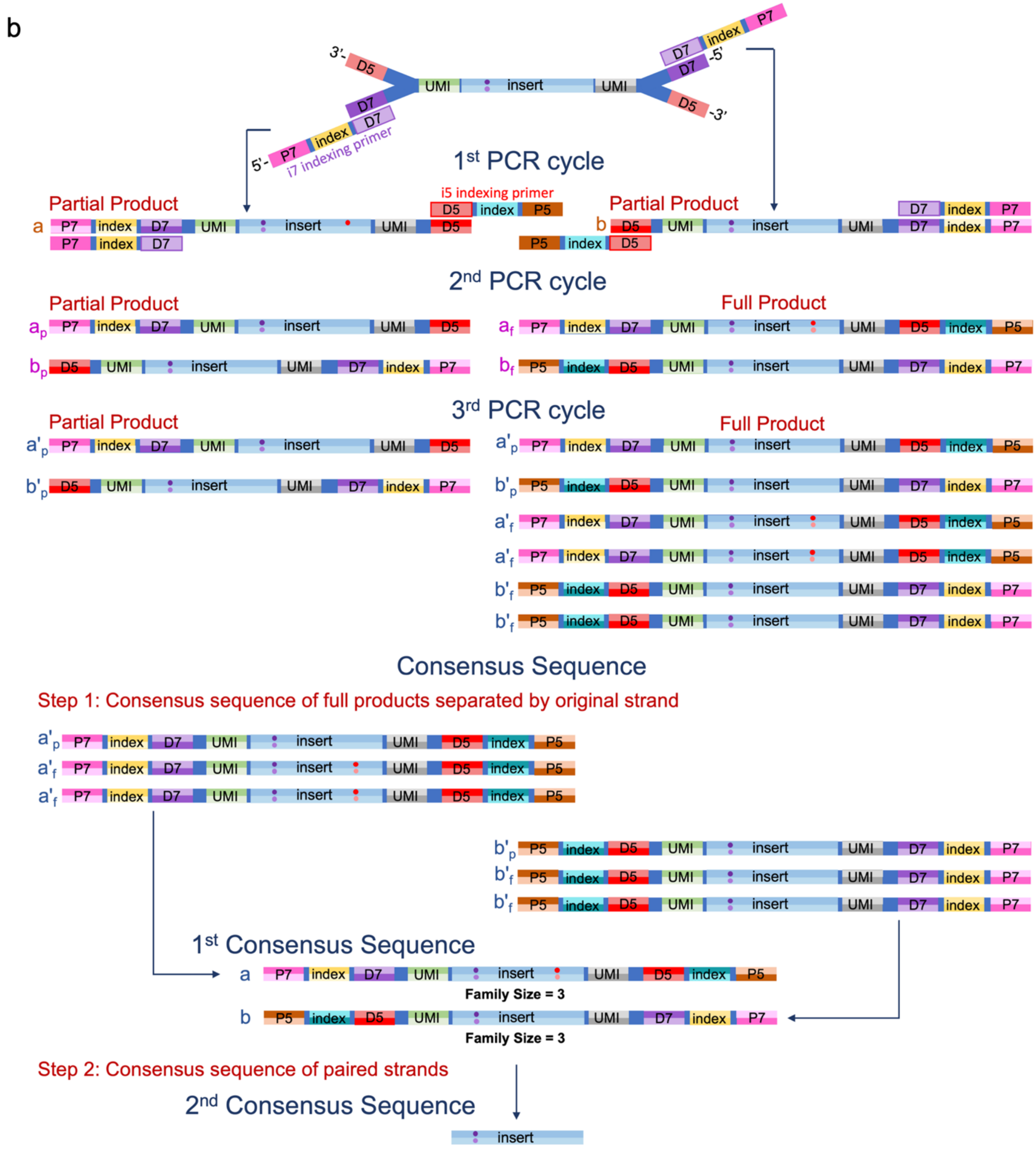

S2 Fig. Schematic for duplex adapters. In (**a**), the sequence for the complete unligated duplex adapter is shown. The dual UMIs (3 bp) are embedded within the short double-stranded

segment of the adapter, while the single stranded segments consist of the duplex primer locations (D7 and D5). The ‘T*G’ denotes a phosphorothioate bond. The duplex primers (i7 indexing primers, i5 indexing primer) are shown with colored matched sequences in the adapter. The primers contain the indices and the P7 and P5 primer recognition sites, which have been colored to match the sequences shown in S1 Fig. In (**b**), the template DNA associated with the primer represents either the primer recognition site (darker coloring) or the primer sequence (lighter coloring). During the first cycle of PCR, only the D7 primer is used for amplification, which yields two amplicons both harboring the same two UMIs, a single index, and the P7 primer sequence (lighter color). Because the second index and the P5 primer sequence has not been added yet, these are referred to as ‘partial products.’ The D5 recognition site is generated during the first cycle of PCR allowing for both D7 and D5 primers to be used in subsequent PCR cycles. Amplification with the D5 primer adds the second index and the P5 primer sequence (lighter color). With each subsequent PCR cycle, both a ‘partial product’ and a ‘full product’ are generated. The partial product results from amplification of previous partial products with the D7 primer. The full product is derived from amplification of a partial product with the D5 primer or a full product with either the D7 or D5 primer. Consensus sequence determination is a two-step process. First (Step 1), all aligned molecules with the same UMI are collapsed into a single sequence. During this initial step, molecules from the two original strands are not combined because the order of the UMI at the 3mer level from different strands is different even though the bases are the same. Specifically, if the UMI for strand A is ‘abc-def’ then the UMI for strand B is ‘def-abc.’ Next (Step 2), the UMI ordering is used to identify aligned paired strands that then undergo a second consensus sequence determination. Theoretically, the use of paired strand information enables removal of early PCR errors. As before, a true variant (purple dots) is shown to amplify consistently. The introduction of a PCR error (red dots) during the first cycle of PCR does not generate a false positive in a consensus sequence compared to the singleton adapters because of the second consensus sequence determination. However, duplex adapters are vulnerable to false positives from PCR errors if paired strand sequence data is infrequent. In this study, paired strand sequence data was present for <0.2% of consensus reads. Thus, all results for the duplex adapters are based on the Step1 consensus sequences.
