# Supplementary material for: The stochastic nature of errors in next-generation sequencing of circulating cell-free DNA
Source: PLoS One. 2020 Feb 21;15(2):e0229063. doi: 10.1371/journal.pone.0229063 (PMC7034809; doi:10.1371/journal.pone.0229063)
Supplement: S3 Fig — For both blunt-ended DNA (a) and A-tailed DNA (b) the ligation efficiency was significantly greater with the duplex adapters than the singleton adapters. On low-input ccfDNA from patients with pancreatic ductal adenocarcinoma (c), a significant difference between ligation protocols was observed. For all reported findings in this study, protocol 1 was used. Procedures associated with protocol 2 were identical except as indicated by the manufacturer’s instructions specific to the different ligation kit that was tested. (PDF) [file pone.0229063.s006.pdf]

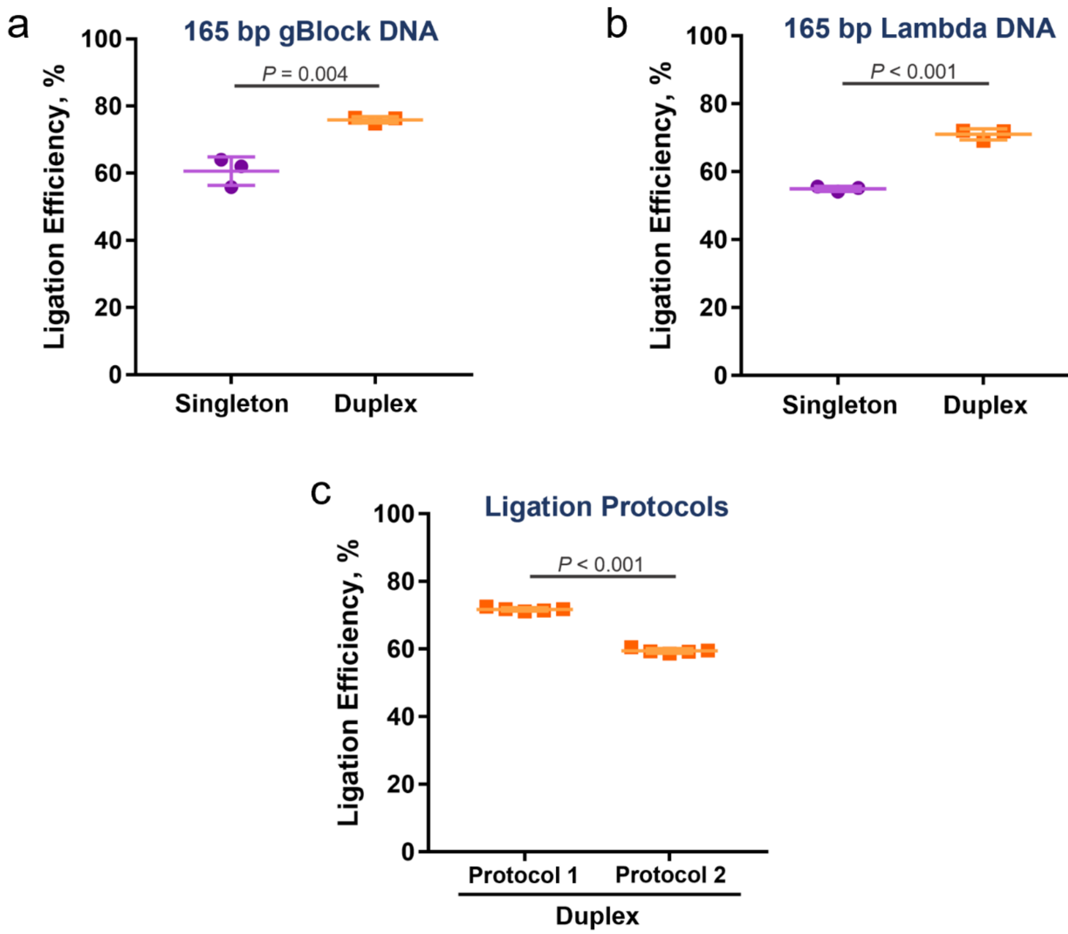

S3 Fig. Ligation efficiency. For both blunt-ended DNA (**a**) and A-tailed DNA (**b**) the ligation efficiency was significantly greater with the duplex adapters than the singleton adapters. On low-input ccfDNA from patients with pancreatic ductal adenocarcinoma (**c**), a significant difference between ligation protocols was observed. For all reported findings in this study, protocol 1 was used. Procedures associated with protocol 2 were identical except as indicated by the manufacturer's instructions specific to the different ligation kit that was tested.
