# Supplementary material for: The stochastic nature of errors in next-generation sequencing of circulating cell-free DNA
Source: PLoS One. 2020 Feb 21;15(2):e0229063. doi: 10.1371/journal.pone.0229063 (PMC7034809; doi:10.1371/journal.pone.0229063)
Supplement: S4 Fig — The error prior to using UMIs for generation of consensus sequences was higher for the duplex adapters compared to the singleton adapters. Bar and whiskers represent mean±SD. (PDF) [file pone.0229063.s007.pdf]

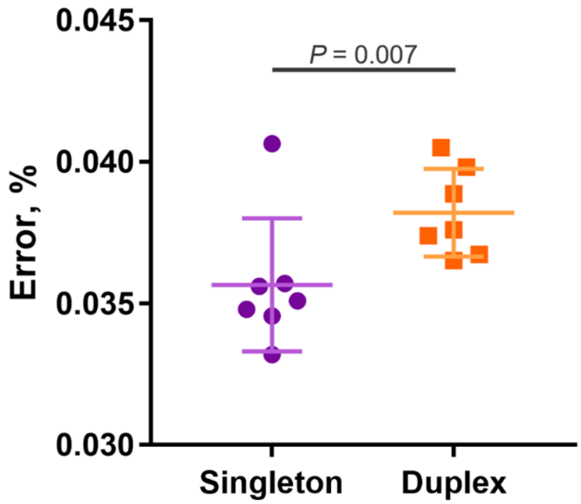

S4 Fig. Error prior to use of UMIs. The error prior to using UMIs for generation of consensus sequences was higher for the duplex adapters compared to the singleton adapters. Bar and whiskers represent mean±SD.
