# Supplementary material for: The stochastic nature of errors in next-generation sequencing of circulating cell-free DNA
Source: PLoS One. 2020 Feb 21;15(2):e0229063. doi: 10.1371/journal.pone.0229063 (PMC7034809; doi:10.1371/journal.pone.0229063)
Supplement: S5 Fig — Nonreference alleles (NRAs) in buffy coat DNA (family size ≥2) with an allele frequency between 2% and 30% were identified in each sample and then graphed based on allele frequency and occurrence in other buffy coat DNA samples. Potential CHIP-related artifacts were present in four out of seven samples. The allele frequencies for each potential CHIP-related variant associated with each patient is shown (triangles, circles, and squares–similar symbols are from the same sample). If present in more than one sample, the NRA frequency is displayed from a single sample. (PDF) [file pone.0229063.s008.pdf]

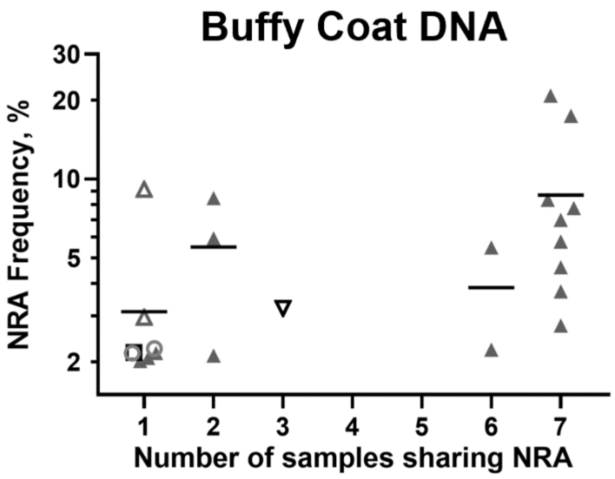

S5 Fig. Potential CHIP-related artifacts. Nonreference alleles (NRAs) in buffy coat DNA (family size ≥2) with an allele frequency between 2% and 30% were identified in each sample and then graphed based on allele frequency and occurrence in other buffy coat DNA samples. Potential CHIP-related artifacts were present in four out of seven samples. The allele frequencies for each potential CHIP-related variant associated with each patient is shown (triangles, circles, and squares – similar symbols are from the same sample). If present in more than one sample, the NRA frequency is displayed from a single sample.
