# Supplementary material for: The stochastic nature of errors in next-generation sequencing of circulating cell-free DNA
Source: PLoS One. 2020 Feb 21;15(2):e0229063. doi: 10.1371/journal.pone.0229063 (PMC7034809; doi:10.1371/journal.pone.0229063)
Supplement: S6 Fig — Note the contribution of CHIP artifacts to noise is relatively small in these control samples. Data points represent the mean value from the seven control samples. (PDF) [file pone.0229063.s009.pdf]

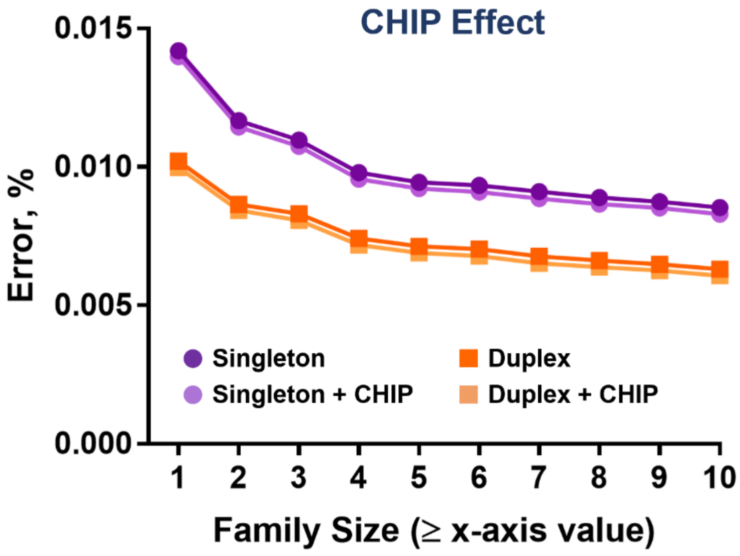

S6 Fig. Effect of removing noise due to CHIP artifacts from singleton and duplex adapters at different family sizes. Note the contribution of CHIP artifacts to noise is relatively small in these control samples. Data points represent the mean value from the seven control samples.
