# Supplementary material for: The stochastic nature of errors in next-generation sequencing of circulating cell-free DNA
Source: PLoS One. 2020 Feb 21;15(2):e0229063. doi: 10.1371/journal.pone.0229063 (PMC7034809; doi:10.1371/journal.pone.0229063)
Supplement: S7 Fig — Nonreference alleles (NRAs) in ccfDNA (family size ≥2) were identified in each sample and then graphed based on allele frequency and occurrence in other ccfDNA samples for both singleton (a) and duplex (b) adapters. The distribution of allele frequencies is from a single sample (the gray triangles correspond to the sample represented with gray triangles in S5 Fig). (PDF) [file pone.0229063.s010.pdf]

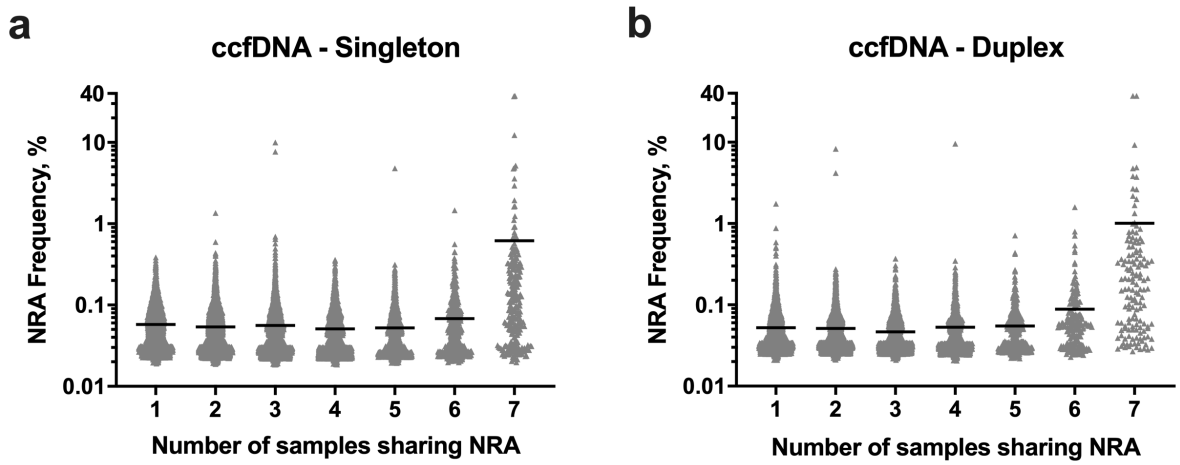

S7 Fig. Patterned error in ccfDNA. Nonreference alleles (NRAs) in ccfDNA (family size ≥2) were identified in each sample and then graphed based on allele frequency and occurrence in other ccfDNA samples for both singleton (**a**) and duplex (**b**) adapters. The distribution of allele frequencies is from a single sample (the gray triangles correspond to the sample represented with gray triangles in S5 Fig).
