# Supplementary material for: The stochastic nature of errors in next-generation sequencing of circulating cell-free DNA
Source: PLoS One. 2020 Feb 21;15(2):e0229063. doi: 10.1371/journal.pone.0229063 (PMC7034809; doi:10.1371/journal.pone.0229063)
Supplement: S8 Fig — In (a) and (b), the original error rate for different family sizes is shown as the top line for singleton and duplex adapters, respectively. Each subsequent line represents the reduction in noise due to removing locations with patterned error. As the sequential lines lighten in color, relatedness is reduced. For example, the first line indicates the error rate when locations are removed that have error in all seven samples. The next line indicates the error rate when locations are removed with error in at least six of the samples and so forth. The bottom line represents the error rate when locations are removed when error is present at a location in two or more samples. The rise in error seen at the lower degrees of relatedness at increment family sizes is due to the greater effects of noise elimination through consensus sequence determination rather than patterned error removal. Although the error rate is progressively reduced, note the effect on the panel footprint at family size ≥2 (c). The more lenient criteria used to define patterned error results in a progressively reduced panel footprint leaving fewer positions available for subsequent analysis. However, in both singleton (d) and duplex (e) adapters the reduction in panel size associated with using reduced relatedness can be mitigated by using larger family sizes. Data points in all figure elements represent the mean value from the seven control samples. (PDF) [file pone.0229063.s011.pdf]

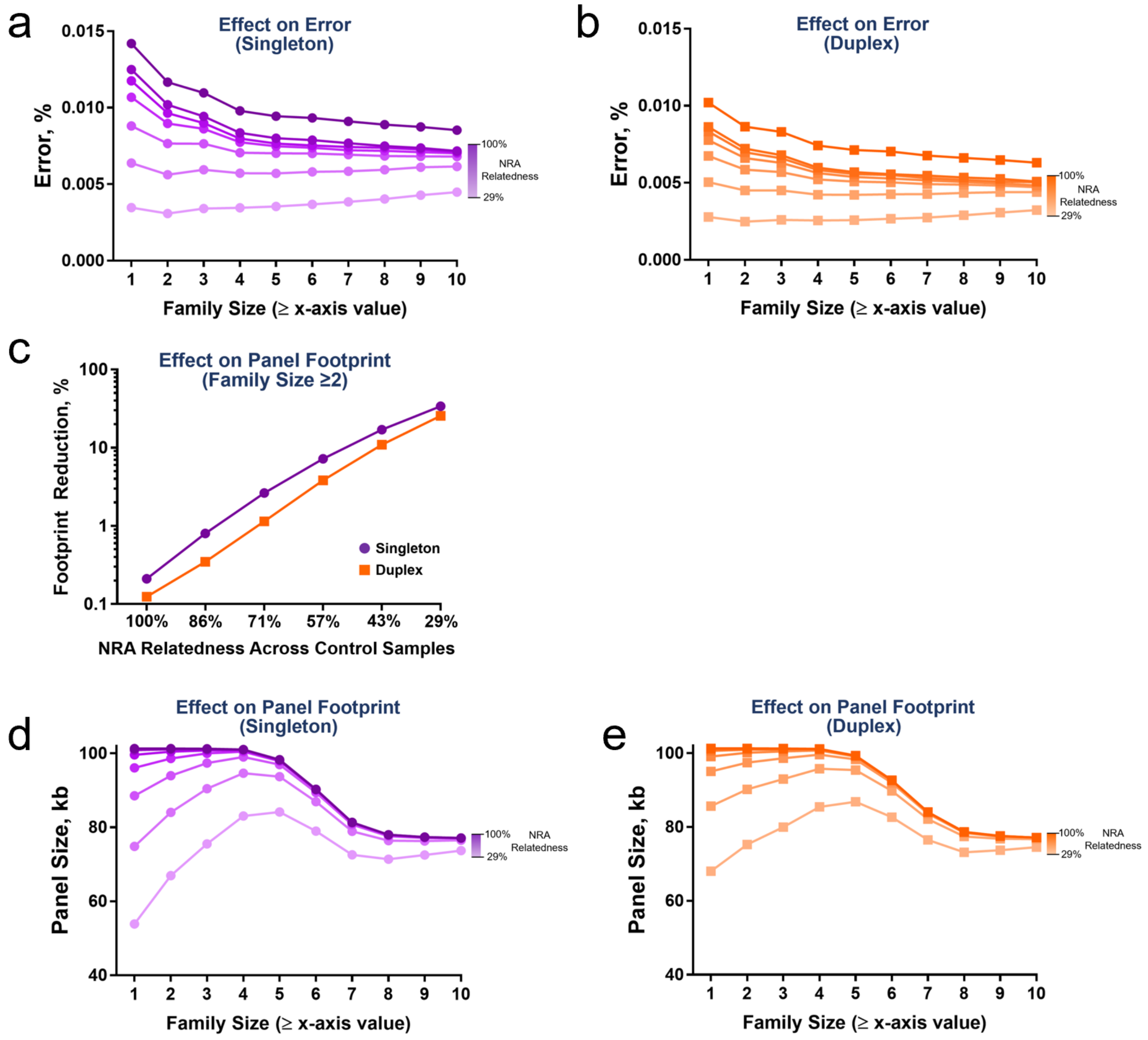

S8 Fig. Effects of patterned error on noise level and footprint size. In (**a**) and (**b**), the original error rate for different family sizes is shown as the top line for singleton and duplex adapters, respectively. Each subsequent line represents the reduction in noise due to removing locations with patterned error. As the sequential lines lighten in color, relatedness is reduced. For example, the first line indicates the error rate when locations are removed that have error in all seven samples. The next line indicates the error rate when locations are removed with error in at least six of the samples and so forth. The bottom line represents the error rate when locations are removed when error is present at a location in two or more samples. The rise in error seen at the lower degrees of relatedness at increment family sizes is due to the greater effects of noise elimination through consensus sequence determination rather than patterned error removal. Although the error rate is progressively reduced, note the effect on the panel footprint at family size ≥2 (**c**). The more lenient criteria used to define patterned error results in a progressively reduced panel footprint leaving fewer positions available for subsequent analysis. However, in both singleton (**d**) and duplex (**e**) adapters the reduction in panel size associated with using reduced relatedness can be mitigated by using larger family sizes. Data points in all figure elements represent the mean value from the seven control samples.
