# Supplementary material for: The stochastic nature of errors in next-generation sequencing of circulating cell-free DNA
Source: PLoS One. 2020 Feb 21;15(2):e0229063. doi: 10.1371/journal.pone.0229063 (PMC7034809; doi:10.1371/journal.pone.0229063)
Supplement: S9 Fig — All data shown are from duplex adapters. ‘Before’ represents the NGS error rate in ccfDNA from the second full library generated with duplex adapters (orange squares). ‘Seq’ represents the error rate associated with sequencing the same capture-enriched library twice. ‘Full’ represents the error rate associated with generation of a full sample duplicate through an independent library formation (the values are the same as shown in Fig 3c, Duplex 2). For both ‘Seq’ and ‘Full,’ error is defined as the same NRA occurring in both corresponding duplicates. Note the substantial reduction in error associated with production of a full sample duplicate compared to sequencing the same library twice. This observation is consistent with early and random PCR errors during library formation being a principal source of noise in NGS. (PDF) [file pone.0229063.s012.pdf]

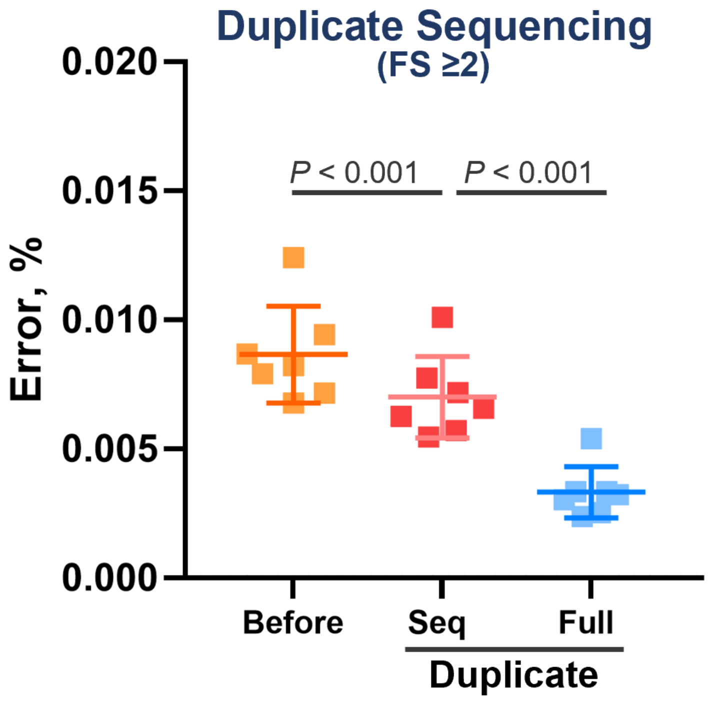

S9 Fig. Effect of a sequencing duplicate compared to a full sample duplicate on error. All data shown are from duplex adapters. 'Before' represents the NGS error rate in ccfDNA from the second full library generated with duplex adapters (orange squares). 'Seq' represents the error rate associated with sequencing the same capture-enriched library twice. 'Full' represents the error rate associated with generation of a full sample duplicate through an independent library formation (the values are the same as shown in Fig 3c, Duplex 2). For both 'Seq' and 'Full,' error is defined as the same NRA occurring in both corresponding duplicates. Note the substantial reduction in error associated with production of a full sample duplicate compared to sequencing the same library twice. This observation is consistent with early and random PCR errors during library formation being a principal source of noise in NGS.
