# Supplementary material for: The stochastic nature of errors in next-generation sequencing of circulating cell-free DNA
Source: PLoS One. 2020 Feb 21;15(2):e0229063. doi: 10.1371/journal.pone.0229063 (PMC7034809; doi:10.1371/journal.pone.0229063)
Supplement: S10 Fig — The initial error after using sample duplicate data (D) is shown for each duplex adapter group at family size (FS) ≥2. Sources of noise, singularly and in combination, were then removed from the duplicate sample data to determine effects on error. Overall, the mean values were significantly different within each sample duplicate–Duplex 1 (F(1.635,9.810) = 146.252, P < 0.001) and Duplex 2 (F(1.381.8.287) = 122.815, P < 0.001). Statistically significant differences between each group within each duplicate are indicated in the figure. Removing CHIP artifacts (+C) had a minor effect. In contrast, removing patterned error (+P) substantially reduced the overall error associated with duplex sample duplicates. The error rate reduction associated with accounting for both CHIP artifacts and patterned error (+C, +P) was largely due to the patterned error contribution. (PDF) [file pone.0229063.s013.pdf]

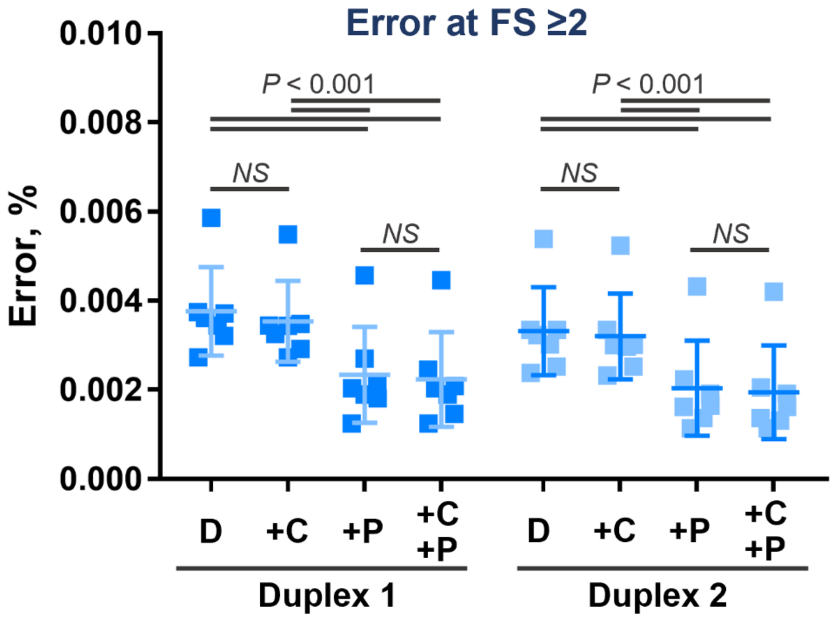

S10 Fig. Effect of removing additional sources of error in duplicate sample data. The initial error after using sample duplicate data (D) is shown for each duplex adapter group at family size (FS) ≥2. Sources of noise, singularly and in combination, were then removed from the duplicate sample data to determine effects on error. Overall, the mean values were significantly different within each sample duplicate – Duplex 1 ($F(1.635,9.810) = 146.252$, $P <$ 0.001) and Duplex 2 ($F(1.381.8.287) = 122.815$, $P < 0.001$). Statistically significant differences between each group within each duplicate are indicated in the figure. Removing CHIP artifacts (+C) had a minor effect. In contrast, removing patterned error (+P) substantially reduced the overall error associated with duplex sample duplicates. The error rate reduction associated with accounting for both CHIP artifacts and patterned error (+C, +P) was largely due to the patterned error contribution.
