# Supplementary figures and images for: The stochastic nature of errors in next-generation sequencing of circulating cell-free DNA
Source: PLoS One. 2020 Feb 21;15(2):e0229063. doi: 10.1371/journal.pone.0229063 (PMC7034809; doi:10.1371/journal.pone.0229063)

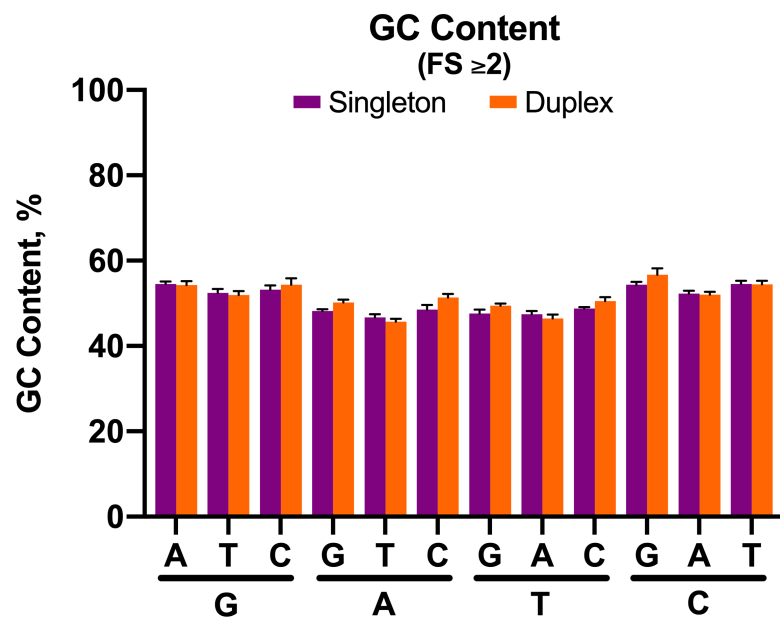

**S11 Fig. Local GC content associated with each type of NRA. FS = family size**

Supplement: S11 Fig — FS = family size. (PDF) [file pone.0229063.s014.pdf]

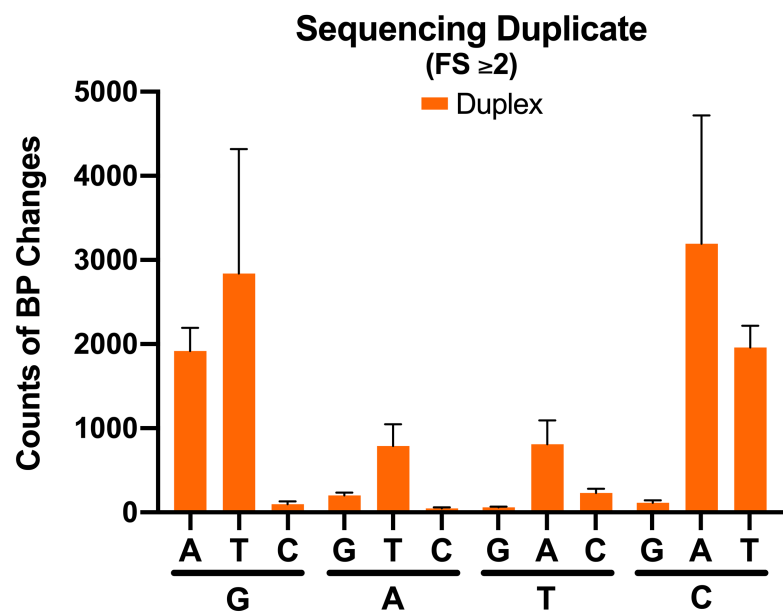

**S12 Fig. Distribution of NRA types present in both sequencing duplicates.** FS = family size

Supplement: S12 Fig — FS = family size. (PDF) [file pone.0229063.s015.pdf]
