# Supplementary material for: The stochastic nature of errors in next-generation sequencing of circulating cell-free DNA
Source: PLoS One. 2020 Feb 21;15(2):e0229063. doi: 10.1371/journal.pone.0229063 (PMC7034809; doi:10.1371/journal.pone.0229063)
Supplement: S13 Fig — In (a), a schematic of the insert (red) and adapter is shown. On densitometry, the adapter migrated at 288 bp (b), a substantial shift from the expected length. This difference is most likely due to the ~56 nt unpaired single-stranded segments which alter the electrophoretic mobility of the adapter compared to double-stranded DNA of a similar length. For example, the peak of the165 bp double-stranded DNA input occurred at the expected size (c). Using (b) and (c) as references, the unligated, single-end, and dual-end ligation products can be identified (d). After PCR (e), the fully double-stranded ligated product (f) occurred at a size consistent with a 165 bp insert and dual-end adapters. (PDF) [file pone.0229063.s016.pdf]

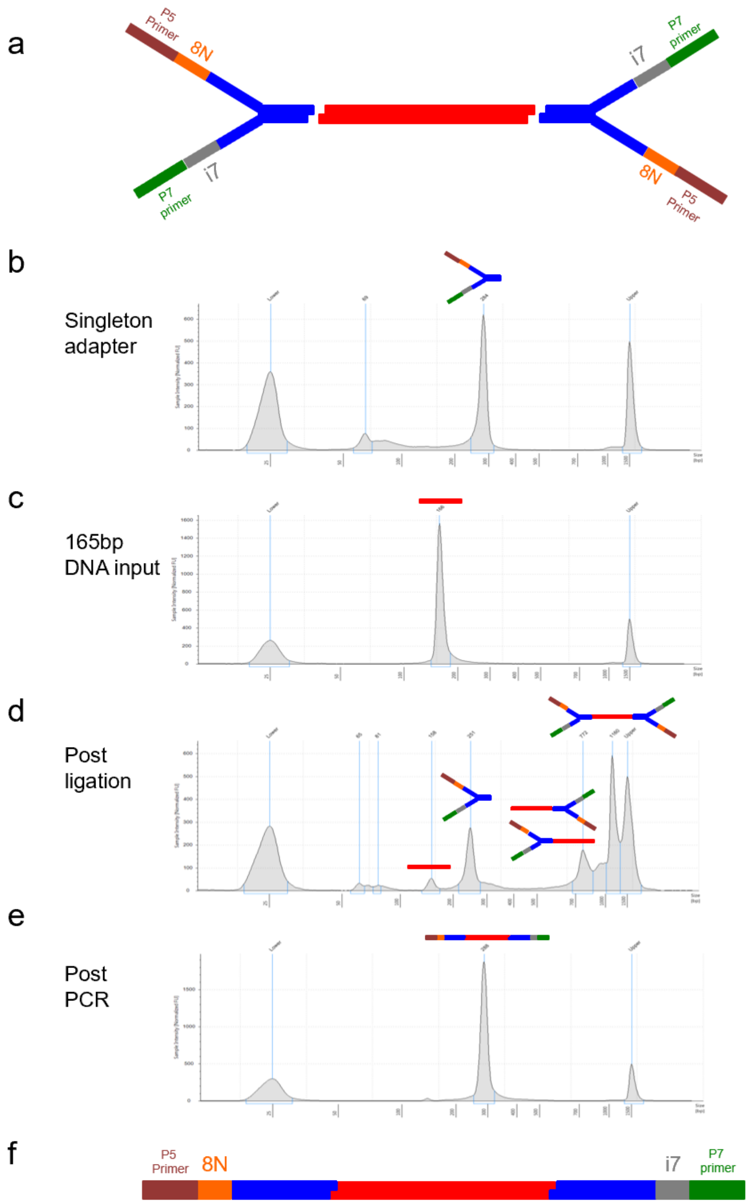

S13 Fig. Densitometry analysis of singleton adapter ligation. In (**a**), a schematic of the insert (red) and adapter is shown. On densitometry, the adapter migrated at 288 bp (**b**), a substantial shift from the expected length. This difference is most likely due to the ~56 nt unpaired single-stranded segments which alter the electrophoretic mobility of the adapter compared to double-stranded DNA of a similar length. For example, the peak of the165 bp double-stranded DNA input occurred at the expected size (**c**). Using (**b**) and (**c**) as references, the unligated, single-end, and dual-end ligation products can be identified (**d**). After PCR (**e**), the fully double-stranded ligated product (**f**) occurred at a size consistent with a 165 bp insert and dual-end adapters.
