# Supplementary material for: The stochastic nature of errors in next-generation sequencing of circulating cell-free DNA
Source: PLoS One. 2020 Feb 21;15(2):e0229063. doi: 10.1371/journal.pone.0229063 (PMC7034809; doi:10.1371/journal.pone.0229063)
Supplement: S15 Fig — The diagram in (a) illustrates the steps in library preparation for adapter ligation. For ddPCR quantitative analysis, a sample was taken after ligation cleanup and after PCR cleanup. For densitometry quantitative analysis (TapeStation), a sample was taken after ligation cleanup. To determine ligation efficiency using ddPCR, two separate reactions were performed. One reaction included a probe for EGFR and a primer pair flanking the probe (internal, b). The second reaction included the same EGFR probe and a primer pair on the adapters flanking the insert (flanking, b). Thus, ligation efficiency was based on the ratio of absolute copy number counts from the flanking primer/probe set (i.e., dual-end ligated) to the internal primer/probe set (i.e., the reference DNA copy number). Ligation efficiency by ddPCR was calculated after ligation cleanup (c). ddPCR measurements after PCR amplification and suppression/removal of any unligated adapters and DNA inserts (d) were performed to show a similar amount of signal from both internal and external primers as indication that the measured ligation efficiency was not principally attributable to differences in PCR efficiency between the internal and external primer pairs. Ligation efficiency by densitometry was done with Tapestation analysis and following ligation clean-up (e). The method for measuring unligated, single-end, and dual-end products is described in S13 and S14 Figs for singleton and duplex adapters, respectively. The ligation efficiency measured by densitometry was similar to that measured by ddPCR (f). (PDF) [file pone.0229063.s018.pdf]

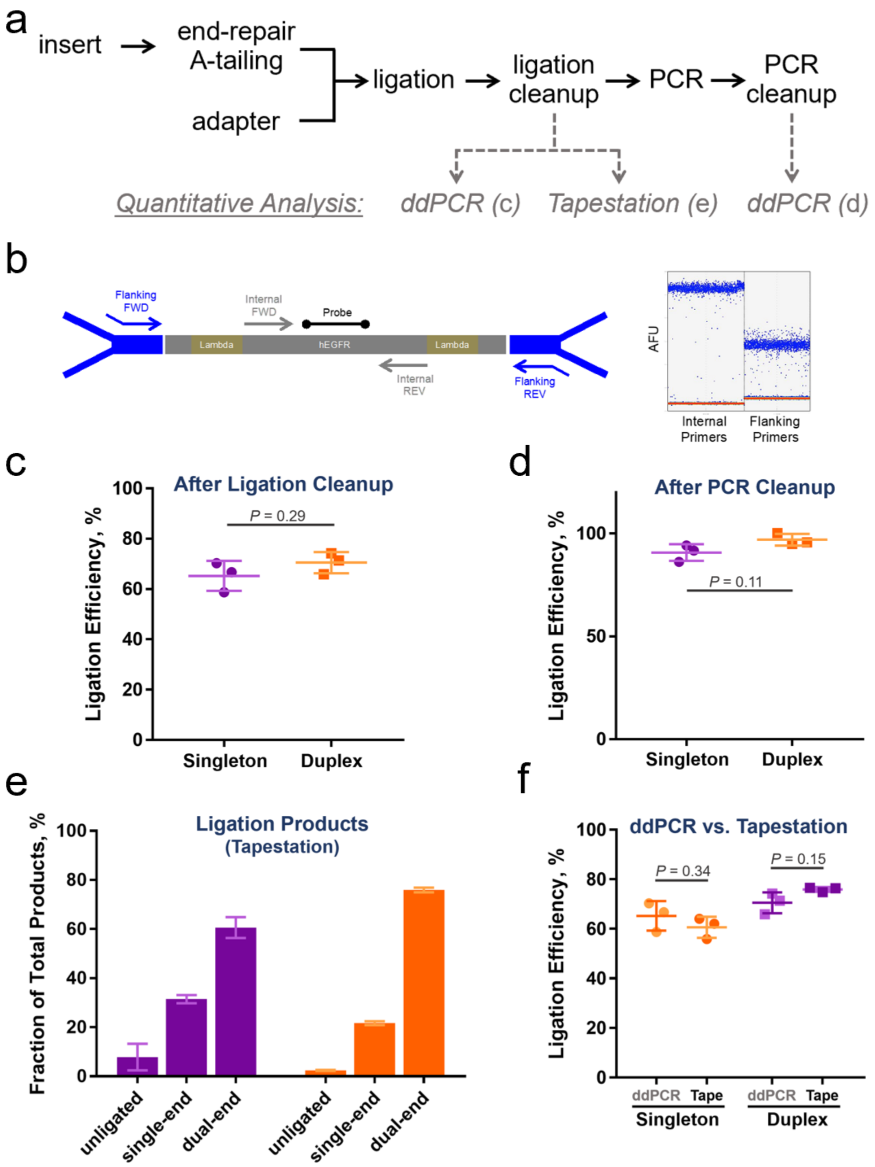

S15 Fig. ddPCR for validating measurements of ligation efficiency with densitometry. The diagram in (**a**) illustrates the steps in library preparation for adapter ligation. For ddPCR quantitative analysis, a sample was taken after ligation cleanup and after PCR cleanup. For densitometry quantitative analysis (Tapestation), a sample was taken after ligation cleanup. To determine ligation efficiency using ddPCR, two separate reactions were performed. One reaction included a probe for *EGFR* and a primer pair flanking the probe (internal, **b**). The second reaction included the same *EGFR* probe and a primer pair on the adapters flanking the insert (flanking, **b**). Thus, ligation efficiency was based on the ratio of absolute copy number counts from the flanking primer/probe set (i.e., dual-end ligated) to the internal primer/probe set (i.e., the reference DNA copy number). Ligation efficiency by ddPCR was calculated after ligation cleanup (**c**). ddPCR measurements after PCR amplification and suppression/removal of any unligated adapters and DNA inserts (**d**) were performed to show a similar amount of signal from both internal and external primers as indication that the measured ligation efficiency was not principally attributable to differences in PCR efficiency between the internal and external primer pairs. Ligation efficiency by densitometry was done with Tapestation analysis and

following ligation clean-up (**e**). The method for measuring unligated, single-end, and dual-end products is described in S13 and S14 Figs for singleton and duplex adapters, respectively. The ligation efficiency measured by densitometry was similar to that measured by ddPCR (**f**).
