# Supplementary material for: The stochastic nature of errors in next-generation sequencing of circulating cell-free DNA
Source: PLoS One. 2020 Feb 21;15(2):e0229063. doi: 10.1371/journal.pone.0229063 (PMC7034809; doi:10.1371/journal.pone.0229063)
Supplement: S16 Fig — The total number of FASTQ reads was similar between singleton and duplex adapters. (PDF) [file pone.0229063.s019.pdf]

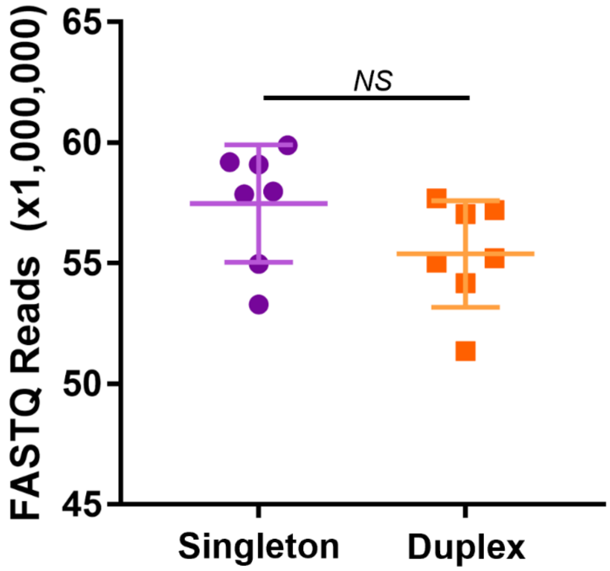

S16 Fig. FASTQ reads. The total number of FASTQ reads was similar between singleton and duplex adapters.
