# Supplementary material for: The stochastic nature of errors in next-generation sequencing of circulating cell-free DNA
Source: PLoS One. 2020 Feb 21;15(2):e0229063. doi: 10.1371/journal.pone.0229063 (PMC7034809; doi:10.1371/journal.pone.0229063)
Supplement: S17 Fig — For family size <5, the panel size is ~101 kb for both the duplex and singleton adapters. Note that the panel size decays rapidly for family size ≥5 as fewer exon positions have consensus reads at larger family sizes. Data points represent the mean value from the seven control samples. (PDF) [file pone.0229063.s020.pdf]

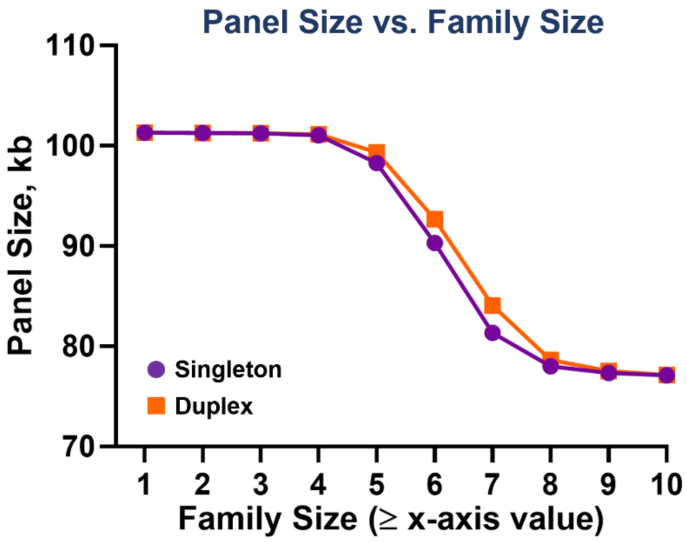

S17 Fig. Panel size based on exon coverage relative to family size. For family size <5, the panel size is ~101 kb for both the duplex and singleton adapters. Note that the panel size decays rapidly for family size ≥5 as fewer exon positions have consensus reads at larger family sizes. Data points represent the mean value from the seven control samples.
